# Exploiting glycan topography for computational design of Env glycoprotein antigenicity

**DOI:** 10.1371/journal.pcbi.1006093

**Published:** 2018-04-20

**Authors:** Wen-Han Yu, Peng Zhao, Monia Draghi, Claudia Arevalo, Christina B. Karsten, Todd J. Suscovich, Bronwyn Gunn, Hendrik Streeck, Abraham L. Brass, Michael Tiemeyer, Michael Seaman, John R. Mascola, Lance Wells, Douglas A. Lauffenburger, Galit Alter

**Affiliations:** 1 Ragon Institute of Massachusetts General Hospital, Massachusetts Institute of Technology and Harvard University, Cambridge, MA, United States of America; 2 Department of Biological Engineering, Massachusetts Institute of Technology, Cambridge, MA, United States of America; 3 Complex Carbohydrate Research Center, Department of Biochemistry and Molecular Biology, The University of Georgia, Athens, Georgia, United States of America; 4 Institute for HIV Research, University Hospital Essen, University Duisburg-Essen, Essen, Germany; 5 Department of Microbiology and Physiological Systems, University of Massachusetts Medical School, Worcester, MA, United States of America; 6 Beth Israel Deaconess Medical Center, Boston, Massachusetts, United States of America; 7 Vaccine Research Center, National Institute of Allergy and Infectious Diseases, National Institutes of Health, Bethesda, MD, United States of America; National University of Singapore, SINGAPORE

## Abstract

Mounting evidence suggests that glycans, rather than merely serving as a “shield”, contribute critically to antigenicity of the HIV envelope (Env) glycoprotein, representing critical antigenic determinants for many broadly neutralizing antibodies (bNAbs). While many studies have focused on defining the role of individual glycans or groups of proximal glycans in bNAb binding, little is known about the effects of changes in the overall glycan landscape in modulating antibody access and Env antigenicity. Here we developed a systems glycobiology approach to reverse engineer the complexity of HIV glycan heterogeneity to guide antigenicity-based *de novo* glycoprotein design. bNAb binding was assessed against a panel of 94 recombinant gp120 monomers exhibiting defined glycan site occupancies. Using a Bayesian machine learning algorithm, bNAb-specific glycan footprints were identified and used to design antigens that selectively alter bNAb antigenicity as a proof-of concept. Our approach provides a new design strategy to predictively modulate antigenicity via the alteration of glycan topography, thereby focusing the humoral immune response on sites of viral vulnerability for HIV.

## Author summary

Carbohydrates on the HIV Env glycoprotein, previously often considered as a “shield” permitting immune evasion, can themselves represent targets for broadly neutralizing antibody (bNAb) recognition. Efforts to define the impact of individual glycans on bNAb recognition have clearly illustrated the critical nature of individual or groups of glycans on bNAb binding. However, glycans represent half the mass of the HIV envelope glycoprotein, representing a lattice of interacting sugars that shape the topographical landscape that alters antibody accessiblity to the underlying protein. However, whether alterations in individual glycans alter the broader interactions among glycans, proximal and distal, has not been heretofore rigorously examined, nor how this lattice may be actively exploited to improve antigenicity. To address this challenge, we describe here a systems glycobiology approach to reverse engineer the complex relationship between bNAb binding and glycan landscape effects on Env proteins spanning across various clades and tiers. Glycan occupancy was interrogated across every potential N-glycan site in 94 recombinant gp120 recombinant antigens. Sequences, glycan occupancy, as well as bNAb binding profiles were integrated across each of the 94-atngeins to generate a machine learning computational model enabling the identification of the glycan site determinants involved in binding to any given bNAb. Moreover, this model was used to generate a panel of novel gp120 variants with augmented selective bNAb binding profiles, further validating the contributions of glycans in Env antigen design. Whether glycan-optimization will additionally influence immunogenicity, particularly on emerging stabilized trimers, is unknown, but this study provides a proof of concept for selectively and agnostically exploiting both proximal and distal viral protein glycosylation in a principled manner to improve target Ab binding profiles.

## Introduction

Env glycoproteins on the surface of enveloped viruses, such as HIV [1–4], Dengue [5, 6], Ebola [7], hepatitis C [8], influenza [9], Lassa [10], and Zika [6, 11], are the primary vaccine targets for the induction of protective, broadly neutralizing antibodies (bNAbs). However, many of these viruses evade the evolution and activity of bNAbs via sequence diversification and the masking of critical Env epitopes by glycosylation. Various molecular engineering approaches have been applied to generate HIV immunogens, such as stablizing a closed conformation of a native like-trimeric Env [12–17] or creating minimal target sites of neutralizing vulnerability on nanoparticluate structures [18]. These efforts have successfully elicited autologous neutralizing antibodies (Abs) in rabbits as well as in macaques [19, 20] and have been shown to guide the first steps of germline bNAb precursor activation [21–26]. Unfortunately, these immunogens have yet to prove sufficient for driving the evolution of broadly cross-neutralizing antibody (Ab) responses [24], indicating that new immunogen engineering strategies are urgently required to improve antigenic profiles of Env immunogens for selective generation of Abs against sites of neutralizing vulnerability.

Glycans represent more than half the mass of the HIV Env glycoprotein, obscuring nearly the entire surface of the Env trimer. While these glycans were originally believed to shield against an Ab response, over the past decade a number of bNAbs have been identified that actively recognize these glycan themselves. Interestingly, these antibodies usually emerge following extensive evolutionary selection enabling them to generate unusual antigen-recognition domains (Fabs) that are able to reach through, and even utilize glycans, to access the underlying protein surface [27–36]. Moreover, through high-resolution imaging approaches, including cryo-electron microscopy (Cryo-EM), it is becoming apparent that bNAb:glycan interactions are common across nearly all bNAb classes. Through linked viral evolution studies in subjects who evolve bNAbs, it is clear that while some glycans are essentially part of the Ab-epitope, other glycans actively block binding, thereby giving rise to a complex network of potential agonists/antagonists [3, 4, 37, 38].

Antigenic opportunities for manipulating glycans on Env were first elucidated in studies focused on either altering the overall glycans or certain glycans specifically [39–43]. These approaches resulted in enhanced antigenicity, as well as the evolution of Abs to sites of neutralizing Ab vulnerability [44]. Removal of particular glycans in the C-terminus of the V2 loop enabled the induction of neutralizing Abs with some breadth in non-human primates (NHPs) [45, 46]. Similarly, the induction of autologous tier 2 neutralization following immunization with native-like SOSIP trimeric Env was reproducibly attributable to induction of nAbs against a “glycan hole” in the shield [47]. Elimination of the glycan located in loop D of the CD4-binding site resulted in enhanced germline B cell activation of VRC01 and NH45-46 precursors [44, 48]. Moreover, recent reports point to the acquisition or loss of particular glycans in driving affinity maturation of specific bNAb lineages [49, 50]. However, while certain glycans may help shape individual epitopes [30, 31, 33, 34, 36, 51], glycans interact with one another, dynamically reshaping the exposed protein surface, coordinately influencing Ab binding access [37, 52–56]. Collectively, these data all highlight the critical role of glycans, as individuals or as groups, contributing to the overall antigenic profile of the Env glycoprotein. Nonetheless, there remains inadequate understanding of systematic principles that could offer an actionable path for the development of immunogens that can exploit the large mass of sugars decorating viral protein surfaces.

While causal relationships between individual glycosylation sites, or groups thereof, and bNAb binding have been defined [27, 30–36, 38, 44–46, 49–56], it is conceivable that modulating glycan interactions more broadly may represent a further means to focus the humoral immune response [57–59]. The Env glycans extend beyond the protein surface and accordingly yield a topographical landscape of the macromolecule, influencing access to the underlying protein. Changes in glycosylation at one site, such as addition or removal of a particular glycan, may have a substantial impact on epitope availability even at relatively distal sites [37, 56]. Therefore, methods that can exploit both the protein and the glycans from a multi-site, landscape perspective, may provide a benefit for improved HIV immunogen design.

In this work we describe a multi-variate, combined experimental/computational glyco-engineering strategy to rationally alter HIV envelope glycosylation with a goal of inducing desirable topographical changes in epitope accessibility. First, a deconvolution model was constructed to reverse-engineer the data in a panel of 94 recombinant HIV Env antigenic profiles screened against a battery of broadly neutralizing and non-neutralizing Abs. Glycan occupancy at each n-linked glycan site (‘sequon’) was assessed by mass spectrometry, and both protein sequence and glycan occupancy were used as variables in the model to elucidate how Ab binding is dependent on them. Next, based on this model, forward-engineering principles aimed at changing the overall antigenic profile of any given HIV Env were ascertained. Using a Bayesian machine learning algorithm permitted determination of key sequon sites that positively or negatively influence Ab binding. Finally, these results were then used in a *de novo*, *in silico* protein antigen design model using an iterative antigenicity-guided sequence evolution framework, seeking to predict alterations in the antigen that could selectively improve or impair target antibody binding. These predictions were then used to synthesize novel antigens that successfully bound to target bNAbs with enhanced and selective antigenicity. All together, our work provides proof-of-concept, in an initial HIV Env application, for the advance of multi-variate glycosite-based engineering of immunogens that can focus the humoral immune response on sites of neutralizing vulnerability.

## Results

### Site-specific glycan occupancy heterogeneity across HIV gp120 strains

Over the past decade, significant effort has been invested in the generation of stabilized, native, trimeric HIV Env proteins or minimal scaffolds of sites targeted by bNAbs, aimed at focusing the immune response to sites vulnerable to antibody mediated neutralization. While exciting results continue to accrue, these proteins alone have not proven sufficient to drive neutralizing Ab breadth. Strategies to further optimize the HIV Env glycoprotein for induction of bNAb breadth thus continue to be urgently needed. Given our emerging appreciation for the antigenic nature of the large array of glycans that decorate the HIV Env glycoprotein, here we hypothesized that the glycan shield itself might be strategically modified and exploited in a rational manner, offering a potentially generalizable glyco-engineering approach that could be applied to either trimeric or monomeric Env proteins. For proof-of-concept purposes, we focused our study on a diverse panel of Env monomers.

**Fig 1:**
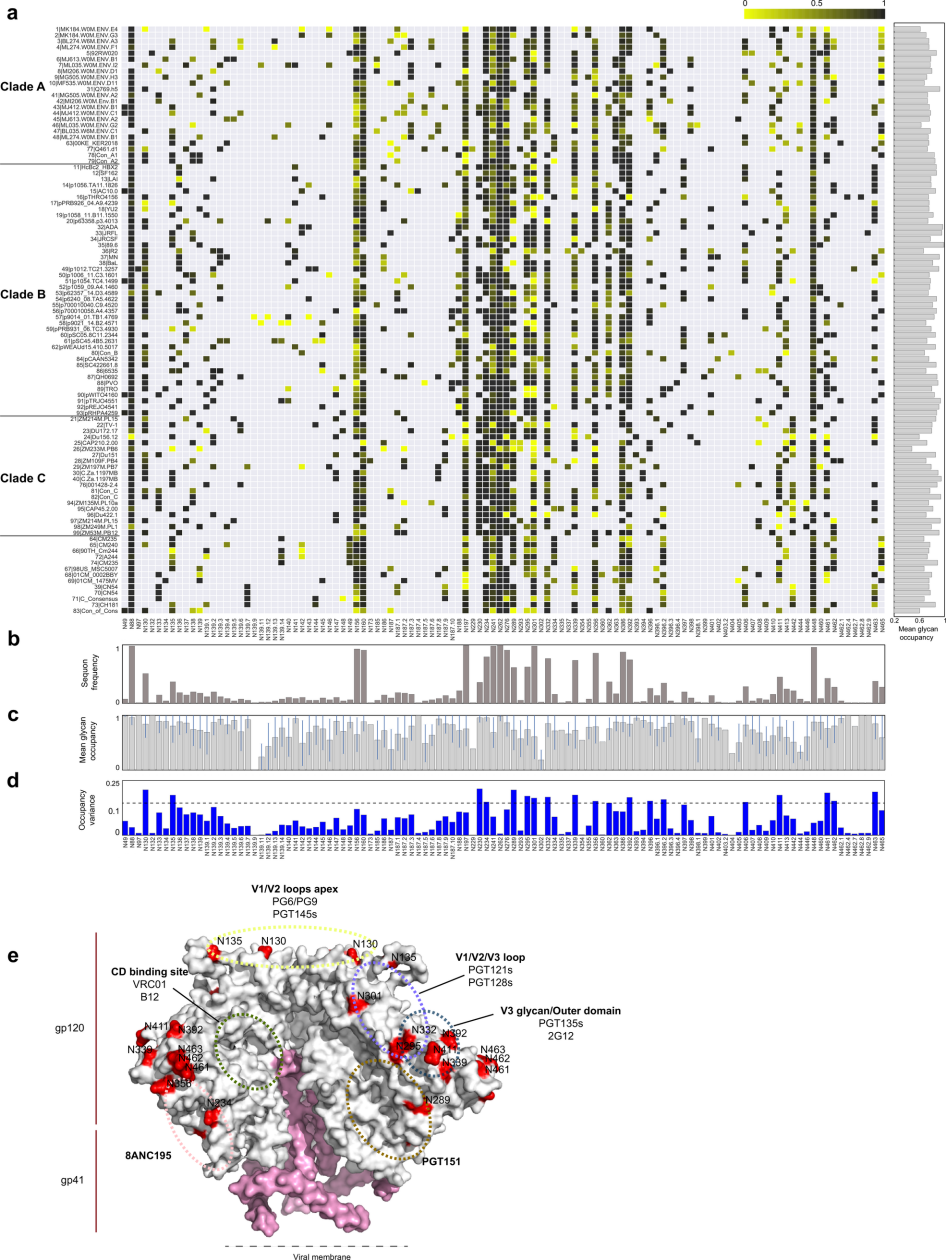
Global glycan occupancy site utilization across 94 HIV gp120s. (a) The heat map represents the N-linked glycosylation site occupancy profiles of 94 distinct recombinant gp120 proteins. Site utilization was determined by mass spectrometry, and the frequency of utilized sites at each potential glycosylation site (columns) is presented using a yellow-to-black gradient. The gray boxes depict the absence of a sequon (N-X-S/T, X≠P) at that specific site within that sequence. The right panel shows the average glycosylation site occupancy per protein. N-glycan sites were aligned based on the HXB2 sequence. Canonical N-glycan sites were designated based on the aligned sequence. Non-canonical N-glycan sites, which are not present in the HXB2 sequence, are shown in decimal numbers, based on the previously aligned N-glycan site. (b)-(e) The bar graphs show (b) the frequency of sequons present at each potential N-glycan site across all strains; (c) the mean (± standard deviation) glycan occupancy; (d) the variance of the glycosylation site occupancy (dotted line represents the top 15th percentile). (e) The N-glycan sites with the top 15% highest variance were mapped onto the BG505.SOSIP crystal structure (PDB #: 4NCO) highlighted as red. The approximate binding epitopes of various bNAbs on the Env structure are labeled in hatched circles.

Because of the remarkable sequence diversity among HIV viral variants, we assessed glycan site occupancy across a panel of recombinant HIV Env monomers (gp120). Traditional assays for HIV Env glycosylation have relied on enumeration of N-linked glycosylation sites (sequons; Asn-X-Ser/Thr, X ≠ Pro), within a sequence (Figure A in S1 File). However, accumulating data suggests that the presence of a sequon is necessary but not sufficient to guarantee the presence of the associated glycan [60–64]. To address this discrepancy, we first conducted a mass spectrometric N-linked glycoproteomic analysis of the glycan occupancy profiles for each of 94 recombinant gp120 proteins that span clades A, B, and C with additional AE, AG, and BC variants (Fig 1, Figure B in S1 File). Site-specific, N-linked glycan occupancy was determined using proteolytic digestion, peptide deglycosylation, occupied site labeling, and MS/MS fragment quantification and site-mapping. The glycan occupancy level was estimated by calculating the ratio of the spectral counts of the glycosylated peptide in relation to the overall incidence of the peptide, where the glycosylated peptide was quantified through ^18^O isotopic labeling [65–67]. To validate linkage of the spectral count to the relative peptide abundance, we tested spectral counting of a pre-mixed peptide population (Table A in S1 File); this peptide mixture contained two nearly identical sequences: one with a particular sequon and another with a Asn-to-Asp substitution within that sequon, to represent glycosylated and non-glycosylated-peptide variants. The ratio of the spectral counts of the two peptides provided consistent quantitation of the peptide relative distributions, giving confidence in this method.

From this N-glycoproteome analysis, 83% of the possible sequons across the panel of 94 gp120 variants were identified as quantitatively present, with 92% of the sequons exhibiting full or partial occupancy (Table B in S1 File). The glycan occupancy profiles strikingly revealed heterogeneity at nearly all sites (Fig 1A, S2 Table), and the likelihood of a site being occupied was independent of its sequon frequency (Fig 1B and 1C, Figure C in S1 File). For example, for some highly-conserved sequon sites (e.g., N156, N197, N241, N301) only moderate glycan occupancy was observed, while other highly-conserved sequon sites demonstrated nearly complete occupancy across the 94 proteins (e.g., N88, N234, N262).

Given the significant variation in viral sequences among the tested gp120s (Figure A in S1 File), the relationship between glycan occupancy and sequon sequence was evaluated to determine whether protein sequence alone could predict sequon occupancy. Classic sequons (N-G-S/T) showed the highest degree of glycan occupancy, whereas N-E-S/T exhibited the lowest glycan occupancy (Figure D in S1 File) and a number of sequons demonstrated intermediate occupancy profiles. We also inspected glycan occupancy variance across all the sites (Fig 1D) and mapped the sites with the top 15% highest variance into a Env trimer structure. Interestingly, sites with high glycan occupancy variance were enriched within known bNAb epitopes (Fig 1E), except for the CD4 binding site epitope. This result indicates that the observed heterogeneity over our 94 proteins is not simply related to unusual glycosylation on monomeric gp120, but rather points to the possibility that HIV may actively exploit variation in both protein sequence and glycan occupancy as a means to escape bNAb detection selectively. In turn, sequons, with reproducibly variable glycosylation, provide an opportunity to engineer glycoproteins.

### Defining the glycan site occupancy relationships to bNAb binding

**Fig 2:**
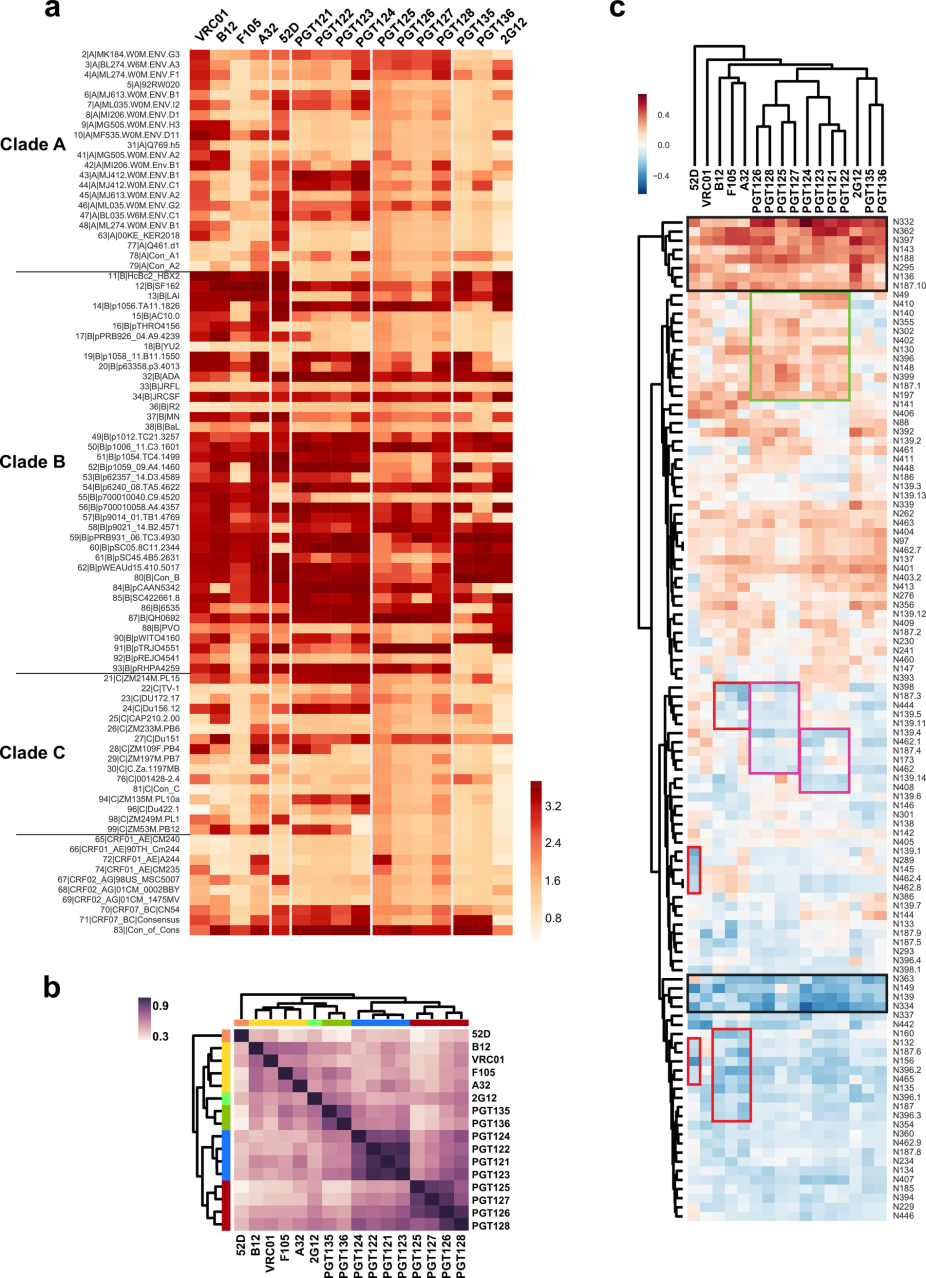
Relationship between glycosylation site occupancy and Ab binding fingerprints. (a) The heat map represents the bNAb- and non-NAb binding profiles for 16 Abs (columns) against a panel of 94 recombinant gp120 glycoprotein variants (rows). The ELISA binding activity was determined by the OD value of the protein concentration = 80 ng/ml based on the protein titration curve (Figure E in S1 File). (b) The correlation matrix illustrates similarities and disparities in the Ab-binding (dark purple = highly correlated; white = non-correlated). Families of bNAbs with more similar glycan footprints cluster along the y-axis or x-axis, depicted by the color blocks. (c) The correlation matrix depicts the relationship between individual Ab binding profiles (columns) and glycosylation site occupancy at individual N-glycan sites (rows) across the gp120 variants. The heat map is color-coded based on the strength and directionality of the correlation coefficient, where antagonistic sites (those decreasing binding affinity) are depicted in blue and agonistic sites (those increasing binding affinity) are depicted in red. The glycan sites defined as “super antagonists/agonists”, which affect binding by all Abs, are highlighted in black squares, and the sites that exhibit epitope-specific modulation against different Ab clusters are highlighted in red, pink, or green squares.

Previous efforts to define the role of glycan occupancy in shaping antibody activity have focused largely on the impact of removing or adding one or a few glycans. We continued our multi-variate approach by using ELISA to interrogate the impact of glycans on Ab binding, for 13 bNAbs and 3 non-NAbs (F105, A32, 447-52D) (Figure E in S1 File) against the 94 gp120s for which glycan occupancy profiling was obtained (Fig 1A). These Abs covered four well-defined epitope clusters: N332 & V3 glycan (PGT121–128), N332 & outer domain glycan (PGT135-136, 2G12), V3 loop (447-52D), and CD4 binding site-like (VRC01, B12, F105, A32) [12, 68]. While all Abs bound to clade B variants more effectively, the various Abs demonstrated unique binding profiles across clade A and C and recombinant variants (Fig 2A). Despite the observed binding profile heterogeneity, clusters of binding profiles were observed across the proteins, pointing to clear epitope families, with clusters of binding including PGT125–128, PGT121–124, PGT135–136, and the CD4 binding site–targeting Abs (B12, VRC01, F105, A32). Thus, related-monoclonals bound preferentially to similar sets of gp120s, as expected based on epitope availability of target epitopes on particular gp120 structures (Fig 2B).

Based on the disparate Ab binding profiles (Fig 2A) and the known glycan occupancy differences (Fig 1A), we ascertained the impact of glycan site occupancy on Ab binding. The correlation matrix, which depicts the pair-wise covariance between glycan site occupancy and Ab binding across the gp120 panel, illustrates a strong relationship between glycan occupancy and Ab binding profiles (Fig 2C). A striking bimodal pattern was observed, where glycan occupancy at specific sites either agonized or antagonized Ab binding. Moreover, intriguing complex patterns also emerged from these data, including the observation of “super antagonist” sites (e.g., N149 and N139) and “super agonist” sites (e.g., N332 and N362; Fig 2C, highlighted in black squares) that affected binding of all tested Abs, potentially acting through glycoprotein stability rather than direct antigenicity. Additionally, sites exhibiting epitope-specific modulatory effects were found (Fig 2C, highlighted in red/green/pink squares). Notably, while the N332 is known for its critical role in shaping V3-family binding, it is not known for its role in shaping other bNAb responses. However, the data here suggest that the N332 glycan plays a much broader role in bNAb binding, including shaping the epitope of all CD4–binding site Abs. This observation indicates that proximal as well as distal glycans can contribute to antigenicity, potentially via alterations in the topographical remodeling of the overall glycan shield. Thus, groups of both positively-influencing and negatively-influencing glycans were associated with individual classes of Abs, pointing to glycans that either selectively decrease binding to non-NAbs (red boxes) along with glycans that either increase or decrease binding to target bNAbs (PGT121 and PGT128 –in green and pink, respectively). These data provide new insights into the directional influence (i.e., agonistic or antagonistic) of individual glycans as well as clusters of glycans in shaping Ab binding to HIV gp120.

### Defining rules that govern the impact of glycan occupancy on bNAb binding using Bayesian machine learning

To more rigorously model the integrative contribution of proximal and distal glycans in collectively shaping Ab binding profiles, and to define the minimal glycans required to shape the overall glycoprotein topology, we developed a supervised Bayesian machine-learning algorithm to capture relationships—linear as well as non-linear—between site occupancy profiles and Ab binding fingerprints. This computational framework consisted of two key parts. First, a support vector regression (SVR) model [69] involving incorporated kernels, to uncover relationships between glycan sites (individually and in combination) and Ab binding. And, second, a Bayesian Markov Chain Monte Carlo (MCMC) sampler [70, 71], to approximate how the various sites and combinations of glycans impact binding and ultimately capture the minimal set of optimal glycan sites that predict binding.

**Fig 3:**
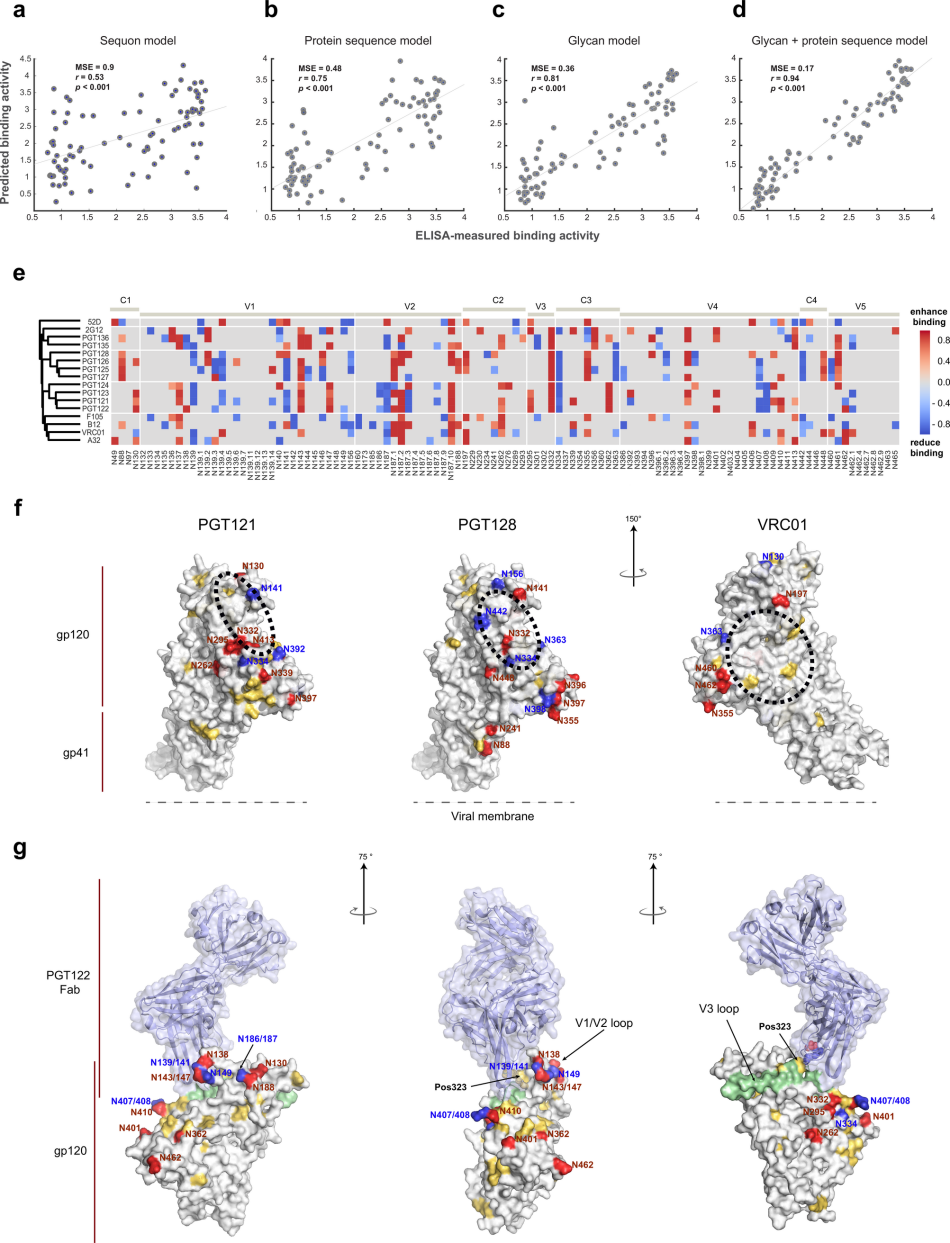
Defining the glycosylation site determinants that shape bNAb binding profiles. (a)-(d) Four different Bayesian MCMC-SVR models were evaluated for their respective abilities to predict PGT121 binding to the 94 proteins. The models include a Bayesian MCMC-SVR model based on: (a) sequon presence (Figure A in S1 File); (b) protein sequence; (c) glycosylation site occupancy; or (d) glycosylation site occupancy and sequence combined. Cross validation (100-iterative 10-fold) was used to evaluate model performance. Goodness-of-fit was assessed and is reported as the mean squared error (MSE) between predicted and ELISA-measured binding. (e) Heat map shows the binding signatures of individual Abs (rows), where the selected glycan sites (determinants) that mediate effects on Ab binding are highlighted. NAbs that share similar glycan determinants are grouped by hierarchical clustering. (f) The significant glycan site determinants for PGT121, PGT128, and VRC01 are plotted onto a 3-dimensional gp120 monomer structure using the same directional color coding as the heat-map. Additionally, the critical protein residues predicted by our model are shaded in yellow on the same 3D structure. Finally, broad Ab-binding sites were highlighted for each bNAb in hatched circles. (g) Agonistic and antagonistic glycan site determinants and critical protein residues for PGT122 are projected on the BG505 SOSIP.664-PGT122 co-crystal structure (PDB #: 4NCO) with the same color coding. The V3 loop is highlighted in light green shading.

As a proof of concept, we applied this approach to defining the minimal combination of glycan predictors for PGT121 binding. We built models based on 4 different types of input variables: sequons alone (Figure A in S1 File); protein sequences alone; glycan occupancies alone (Fig 1A); protein sequences and glycan occupancies inputs. 10-fold cross-validation was performed for each model to test robustness and prediction accuracy. The models based on sequons or sequences alone were correlated relatively poorly to the experimental ELISA binding results, with r = 0.53 and 0.75, respectively (Fig 3A and 3B). The model based on glycan occupancy alone was better correlated with binding activity (r = 0.81, *p* < 0.001; Fig 3C), suggesting that glycan occupancy was more predictive that sequence alone in predicting antibody binding. However, the fourth model, combining both glycan occupancy and protein sequence, showed the best performance with robust predictive power (*r* = 0.94, *p* < 0.001) and a very low MSE (0.17) (Fig 3D), confirming that combination of glycan occupancy and sequence information can substantively account for the complex interaction between Abs and Env proteins.

Given the superior improvement in model performance when both glycan occupancy and sequence were included, we next constructed models for additional Abs, incorporating directionality of glycan on antibody binding (i.e., positive or negative influence on binding) to deconvolute and define the specific glycosylation sites (Fig 3E and Figure F in S1 File, *q* < 0.01) and amino acid residues (S3 Table) that most strongly contribute to binding. Hierarchical clustering consistently showed that Abs recognizing related epitopes share similar glycan-utilization profiles (Fig 3E). Glycans previously appreciated to be involved in shaping antibody binding, including N332, were clearly found to be important across all the PGT Abs along with 2G12, but were not among the most critical predictors of binding for models for the CD4bs-dependent Abs. Importantly, critical glycans for all Abs were observed across the entire protein sequence, indicating that glycans proximal as well as distal to the Ab binding site contribute to Ab binding profiles. Nonetheless, it must be considered that our models, based on the limited 94-monomer dataset, may not completely rule out additional sites.

To gain deeper understanding of how proximal and distal glycans influence Ab binding, the linear glycan sites and protein residues were projected onto a 3-dimensional structure of gp120 (Fig 3F, Figure G in S1 File). Using PGT121, PGT128, and VRC01 as examples, both positively-influencing and negatively-influencing glycan sites were found within the PGT121/128 binding site but not within the VRC01 binding site, as expected. However, both categories of glycan sites were also observed outside the immediate Ab binding region for all Abs. Interestingly, critical protein residues defined by the model were located both within and outside the VRC01 epitope but were largely scattered outside the PGT121/128 binding sites. These findings highlight broad distribution of influential glycans and amino acids across the surface of the HIV Env protein beyond those within the Ab binding site itself; we imagine that this influence likely transpires through alterations in the overall topography of the viral protein surface.

Finally, projection of PGT122-specific glycan and amino acid determinants on the co-crystal structure of the PGT122 Fab and BG505 SOSIP gp140 Env trimer [4] further illustrates how these sites coordinately impact Ab binding (Fig 3G). Sites facilitating PGT122 recognition comprised glycans that participate directly in Ab binding (N332 and N295), proximal glycans located on the nearby V1/V2 loops (N130, N138, N143/147 and N188), and distal glycans located on the outer domain of gp120 (N262, N362, N401, N410 and N462) that may reduce conformational flexibility of glycan clustering and maintain epitope accessibility [37, 53]. Conversely, antagonistic glycan sites were mostly located immediately around the binding site (N139/141, N149, N186/187, N334 and N407/408). This arrangement could reshape protein topography to limit epitope access or increase local steric hindrance preventing further glycan processing into the correct glycan structures [53], in either case interfering with PGT122 binding. Additionally, key amino acid determinant, residue 323, was identified within the epitope [4], whereas others were scattered across the protein, highlighting both the proximal and distal influence of both glycans and amino acid changes in shaping the protein surface. Thus, as the majority of crystal structure studies have focused mainly on proximal glycans that contribute directly to Ab access, the importance of distal inhibitory or agonizing glycans may also be exploited to optimize immunogen design.

### Using topography oriented rational design to develop antigenically enhanced gp120 glycoproteins

**Fig 4:**
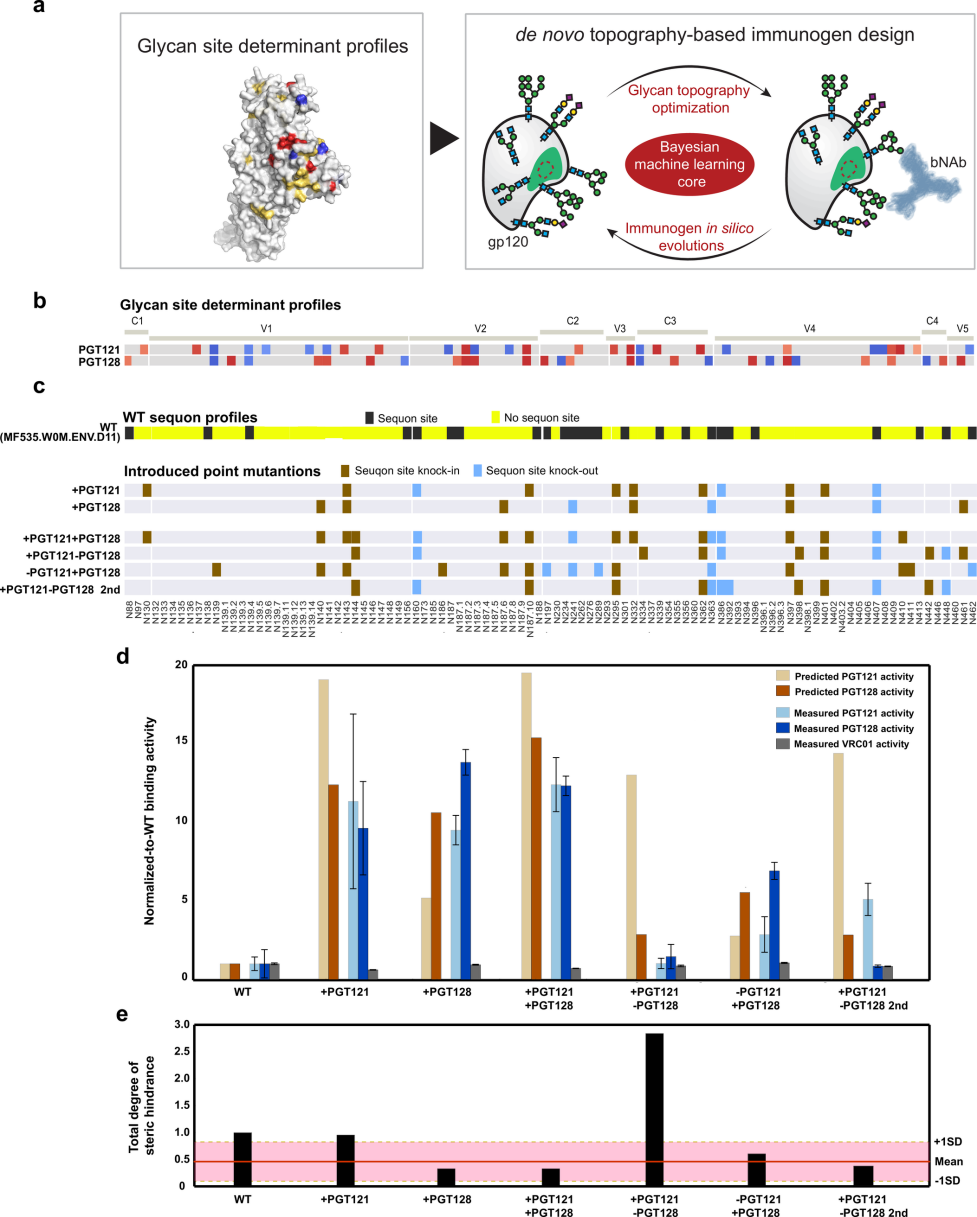
Proof-of-concept glycoengineering of gp120 antigens to selectively enhance antigenicity. (a) Cartoon depicts the overall *de novo* antigen optimization design approach. (b) The heat maps, as in Fig 3E, depict the glycosylation site determinant profiles preferred by PGT121 and PGT128 including directional glycan coloring across all N-glycan sites (columns). (c) The top heat map represents the original wild-type MG535.W0M.ENV.D11 gp120 sequon site profile (yellow = sequon site absent and black = sequon site present); middle and bottom heat maps indicated the introduced point mutations (brown = sequon site knock-in, light blue = sequon site knock-out) for the gp120s engineered to have increased binding to PGT121 (+PGT121), PGT128 (+PGT128), and both PGT121 and PGT128 (+PGT121+PGT128); also, the gp120s engineered to selectively bind PGT121 but not PGT128 (+PGT121-PGT128), or PGT128 but not PGT121 (-PGT121+PGT128 and -PGT121+PGT128 2nd). (d) The bar graph depicts comparison of the predicted binding (beige = PGT121 and brown = PGT128) and ELISA-determined binding (light blue = PGT121, dark blue = PGT128, and grey = VRC01 binding) to the wildtype and engineered gp120s. ELISA binding activity was determined as in Fig 2A. In order to compare the model predictions to the experimental results, both the model and actual ELISA values were normalized to wild-type binding values, which were set to 1. Error bars indicate the standard deviation from six replicates. (e) The bar graph shows the degree of steric hindrance found on each antigen by summing all steric glycan site pairs (Figure J in S1 File), if any site in the pair was considered essential for predicting Ab binding. Pink highlighted region denotes the average and range of the degree of steric hindrance across all the 94 recombinant gp120 proteins.

With an appreciation for the contribution of both proximal and distal glycans in shaping Ab binding, we hypothesized that glycan occupancy signatures could be exploited to guide the design of gp120 proteins with improved binding properties for specific bNAbs. We employed an optimization algorithm for this purpose, based on dynamic evolution based on glycan occupancy, using the same Bayesian machine learning core as above. This algorithm iteratively evolved a given gp120 sequence *in silico* by perturbing defined individual glycan sites (mutating one glycan site at a time), aimed at driving enhanced or impaired bNAb binding for a given bNAb (Fig 4A). This serial computational mutagenesis approach sampled the antigenicity landscape of any given combination of glycan occupancy profiles, ultimately generating a particular combination of glycans that could enhance or impair target bNAb binding most effectively. During iterative glycan site perturbations, the model assumes that the changes (e.g., knock-in or knock-out) of target sites do not influence glycan occupancy of other sites. This approach was applied with the goal of generating immunogens optimized for PGT121 and/or PGT128 binding, due to their related but distinct antigenic fingerprint profiles (Fig 2A). The clade A MF535.W0M.ENV.D11 gp120 sequence was used as a starting sequence, because of its negligible binding profile to both bNAbs (Fig 2A, Figure E in S1 File).

Antigens were first designed to exhibit enhanced binding to one of these two bNAbs, based on the addition of bNAb-specific agonistic glycan sites and removal of bNAb-specific antagonistic glycan sites (Fig 4B and 4C, +PGT121 and +PGT128). The engineered proteins were expressed (Figure H in S1 File) and proper folding was verified based on binding by VRC01 (Figure I in S1 File). In successful validation of our model predictions, both antigens exhibited enhanced binding to their respective bNAb: PGT128 binding was enhanced to the +PGT128 antigen, and PGT121 binding was enhanced to the +PGT121 antigen (Fig 4D and Figure I in S1 File). We also engineering a third antigen, with the goal of concomitantly optimizing glycosylation to enhance binding to both PGT121 and PGT128 bNAbs (Fig 4B and 4C, +PGT121+PGT128). The resulting +PGT121+PGT128 antigen showed significant enhancement of binding activity (12-fold increase) to both bNAbs (Fig 4D). These results argue that our antigen design approach based on the addition or removal of both distal and proximal agonistic or antagonist glycans, aimed at remodeling the overall topography of HIV Env antigens, can effectively improve antigenicity.

As a yet further design challenge, we tested whether glycan engineering could selectively shift antigenicity between bNAbs–facilitating access to one particular bNAb while occluding another. Toward this aim, we designed antigens predicted to enhance PGT121 binding while inhibiting PGT128 binding or vice versa (Fig 4B and 4C, +PGT121-PGT128 or -PGT121+PGT128), despite the propinquity of the PGT121 and PGT128 epitopes. As an example for the +PGT121-PGT128 antigen, PGT128-specific antagonists such as N442 were added while PGT128-specific agonist glycan sites such as N140 were removed or kept absent. Because several glycan sites exist that impair binding of both bNAbs, decisions about which sites to include were dictated by the quantitative degree of a specific glycan contribution to individual bNAb binding models. For instance, N139 had a negative influence on binding for both PGT121 and PGT128, but this effect was stronger for PGT121 than PGT128 (Fig 4B, top panel); accordingly, N139 was incorporated into the -PGT121+PGT128 antigen. The–PGT121+PGT128 antigen in fact demonstrated selective binding to PGT121 and PGT128, to levels even higher than predicted; however, selective binding was not observed for the +PGT121-PGT128 antigen. While the algorithm successfully generated an antigen that was selectively recognized by PGT128, we sought to understand why the antigen predicted to be PGT121-selective failed (Fig 4D).

Closer inspection of the glycan sites in +PGT121-PGT128 showed that a cluster of tightly packed glycans (N339, N355, N362, N363, N392, N396 N398) were chosen by the model, and we recognized that these could have adverse effects on PGT121 binding. To test the corollary hypothesis, then, that these new glycans may have created glycan clusters that sterically hindered Ab binding or unstabilized gp120 conformation (Figure J in S1 File), we estimated their steric effects using glycan-glycan auto-correlations based on occupancy profiles. Relative to the total degree of steric hindrance observed for the MF535.W0M.ENV.D11 gp120 sequence, which was approximately 1 (Fig 4E), the overall degree of glycan steric effects across all 94 gp120s was relatively similar and homogenous across all sequences (Fig 4E, pink region). This level of steric effects between glycans likely reflects a natural fitness landscape of the gp120 glycan density profiles that are naturally accommodated across clades and tiers of viruses. Similarly, glycan steric effects of the novel antigens, other than the +PGT121-PGT128 antigen, were close to those observed for the panel of gp120 proteins. On the other hand, the degree of steric hindrance observed for the +PGT121-PGT128 construct was significantly higher than all other antigens—nearly three times the degree of steric hindrance observed for the original MF535.W0M.ENV.D11 sequence (Fig 4E). Thus balancing steric effects, in addition to optimizing individual glycans, likely offers the optimal approach to modulate antigenicity.

Based on this new insight, we designed the +PGT121-PGT128 antigen taking into consideration the mutual steric effects of glycans as an additional design principle. Reducing glycan steric hindrance while preserving the glycan that could concomitantly agonize PGT121 antagonize PGT128, a new +PGT121-PGT128 2^nd^ was generated. This novel antigen readily bound PGT121 and was not recognized by PGT128 (Fig 4D), in a manner demonstrating good consistency between model predictions and experimental measurements (r = 0.78 in PGT121 binding; r = 0.90 in PGT128 binding prediction, Figure K in S1 File), further validating our modeling approach. More broadly, these results suggest that an expanded analysis of the glycan occupancy profiles of a larger number of non-neutralizing and neutralizing Abs, against not only gp120 but also gp140 and the emerging native-like trimers, could prove beneficial for designing antigens yielding enhanced antigenicity to drive specific desired bNAbs.

## Discussion

With the growing appreciation for the participation of glycans in bNAb binding and the striking effects observed with the removal or addition of individual or groups of proximal glycans [39–45, 48], new antigen design approaches able to exploit these post-translational structures could offer a powerful avenue to improve development of therapeutically effective antigens. Our study aimed to develop a systems glycobiology approach to exploit glycosylation to enhance antigenicity, by integrating global site-specific glycan occupancy, peptide sequence, and Ab binding fingerprints to a panel of 94 distinct HIV envelope monomers, using computational modeling to predict engineered antigens optimized for desired binding characteristics. Our findings demonstrate an extensive heterogeneity in glycan occupancy profiles across 94 distinct HIV Env gp120s, dictated by unique sequons, which together define glycans that shape Ab binding. In addition, glycan mapping across these Env antigens raised a previously underappreciated role of both proximal and distal glycans together, distributed in 3D structures over the glycan surface, that govern Ab binding by potentially shaping the overall surface of the glycoprotein in manner that may alter epitope exposure and access. Moreover, this information was then used to build a computational antigen design algorithm, aimed at optimizing glycan occupancy profiles for desired antigenicity. Engineered antigens successfully enhanced binding to target bNAbs, and even were able to selectively skew antigenicity to discriminate between bNAbs that target overlapping regions of the viral envelope. These predictions were successfully validated by dedicated tests of a number of experimentally produced antigens with respect to binding properties across the set of targeted bNAbs. Of course, whether antigenicity represented by Ab binding will translate to enhanced immunogenicity[12, 19], and whether this approach more generally will apply to native Env trimers or other viral antigens, is uncertain. Nonetheless, our study provides proof of concept that a surface glycoprotein glycan shield can be engineered in a rationale, computation-based manner, to improve target Ab binding profiles.

Analyses of glycan site occupancy on two Env trimers, the BG505 SOSIP.664 [12] and Clade C CZA97.012 [13] trimers (Figure L a-b in S1 File), has pointed to higher overall occupancy on trimeric forms of Env [13, 37, 72, 73] compared to the various monomer sequences examined in this study. However, among these monomers occupancy varied from 63–97% (Fig 1A, right panel), where some sequences exhibited occupancy profiles similar to previously analyzed native trimers, linked to the inclusion of specific high occupancy sequons that also exist within previously studied trimeric antigens. As occupancy is controlled co-translationally and post-translationally [74], differences observed on trimers as well as monomers are likely not only related to misfolding or mis-processing of the proteins but also, critically, to sequence variation (Figure D in S1 File), local secondary structure, as well as surface geometry around the sequons that may all contribute to determining whether Oligosaccharyltransferase (OST) can access a glycosylation site [75]. Moreover, that the highest degree of occupancy variation is observed at N-glycan sites critical for bNAb binding, further argues that the observed variation is not due to aberrations in glycosylation of monomers, but related to evolutionary selection of variability at key sites of viral vulnerability. However, beyond occupancy differences, that are likely less divergent between monomeric and trimeric protein structure, the composition of the glycan may be more highly influenced by quaternary structures due to enzyme accessibility issues. Thus, while only a limited number of trimers have been analyzed for glycan occupancy thus far, with restricted clade and neutralization tier coverage, future analysis of larger numbers of trimers may provide critical insights into the opportunities to glycoengineer more native-like molecules along the lines of our approach.

Protein engineering strategies aimed at generating soluble native-like Env trimers, such as stablilzing the gp120:gp41 interaction [12–15], fixing the epitopes in a closed conformation [16, 17], and optimizing combinations of different trimer variant sequences [76] have been proposed to either target bNAb precursors [21–23] or induce Abs with cross-reactive breadth [19, 20]. We elected here to use a panel of 94 distinct gp120 monomers, due to the breadth of sequences that could be selectively analyzed, to exploit both sequence and glycan diversity in our glycan engineering efforts. Importantly, while nearly all sequons are reproducibly occupied on the SOSIP.BG505 immunogen similar to the previous report [73] (Figure L c in S1 File), preliminary analyses of additional native-like trimers suggest increased variability in glycan occupancy (Figure L a in S1 File), driven by diverging sequons. Despite these differences, the use of a library of monomeric antigens possessing sequence as well as glycan occupancy heterogeneity, gave us a unique opportunity to develop a robust model interrogating the role of individual glycans and sequences in tuning Ab binding profiles. This data set afforded the discovery of novel sequons that can be exploited to control levels of glycan occupancy in the future for HIV and other therapeutic proteins. Moreover, results from our study pointed to a role for distal glycans in shaping Ab binding profiles, emphasizing the need to include information outside of an Ab-binding footprint to develop antigenically enhanced antigens. Given that the glycan shield covers the majority of the Env surface, our glycan engineering approach may provide a novel strategy for vaccine development efforts via the modulation of overall antigen topography to selectively mask immunodominant, and potentially distracting, epitopes, while improving the targeted induction of bNAbs through the creation of targeted “glycan holes” that enable vulnerable site recognition [20, 47]. Additionally, because inter-protomeric glycan:glycan interactions have been observed in the trimeric structure [37, 77], it may be additionally possible to optimize trimer stability or even adapt antigenicity fingerprinting to screen for improvements in germline-reverted B cell receptor recognition to prime immunity more effectively [44, 78].

The MF535.W0M.ENV.D11 gp120 sequence was selected as an initial point for our glycoengineering approach (Fig 4B). While this wildtype gp120 contains N137, N156, and N301, other critical glycans for PGT121 and PGT1218-binding (N295 and N332) are missing [35, 52, 54, 79]. Thus this sequence was optimal for engineering, given its negligible binding to both bNAbs (Fig 2A, Figure E in S1 File). We designed our optimized gp120 proteins using a computational design algorithm in which the selected mutations were focused on the glycan site determinants for both PGT121 and PGT128, including proximal and distal sites (Fig 4B). While some N-glycans, such as the addition of N295 and N332, likely have more profound effects on improving PGT121 and PGT128 binding, addition and removal of other glycans, that were both proximal and distal, also modulated overall as well as selective bNAb binding.

Our computational design model involved a hybrid framework combining machine learning algorithms (support vector machine, SVM) with a Bayesian MCMC sampler. Advantages of integrating these two algorithms were multiple: the SVM implemented kernels capturing mutual dependencies among multiple variants (glycans and protein residues); the Bayesian MCMC sampler reduced computing time and mathematically ensured the identification of the optimal solutions in a high-dimensional feature space; the probabilistic machine learning model then dealt with uncertainties of measurements from high-throughput assays (N-glycoproteome analysis, ELISA-based Ab binding), reducing model overfitting of noisy data; and, the flexible machine leaning enabled the incorporation of additional sequence, glycan structure, neutralization profiles, and affinity measurements, that ultimately led to the generation of a robust model. Future efforts aimed at designing next-generation glyco-engineered immunogens may benefit from a number of further advances including (but not limited to): the incorporation of glycan occupancy data from a larger number of Env proteins (including monomers, trimers, and native-like structures); the inclusion of the glycan structure data itself (e.g., oligo-mannose, hybrid, complex); the addition of binding profiles from a larger repertoire of Abs (including both broadly neutralizing and non-neutralizing Abs), germline binding profiles, measures of binding affinity/avidity; the inclusion of virus:BCR evolutionary dynamics to help the algorithm learn to evolve envelope intermediates; and, the final consideration of occupancy profiles from envelopes on the surface of the virus or the cell, that may adopt distinct conformations.

Given the enormous evolutionary landscape explored by HIV, and the fact that carbohydrates represent half the mass and most of the surface of the viral envelope, our study points to the importance of exploring both glycan occupancy and sequence diversity for optimal antigen design. In the future, our approach may be extended to address profiles associated with enhanced germline BCR binding or intermediate BCR ancestor interactions, ultimately aimed at designing sequential immunization strategies or “multi-valent vaccines” able to elicit multiple lineages of bNAbs from a single “super immunogen”. Ultimately, we believe that our work raises promising prospects for topographically designed optimized glycoproteins not only through protein engineering but also through the remodeling of the glycoprotein glycome, which in concert may maximally enhance immunogen antigenicity.

## Materials and methods

### HIV gp120 variant proteins and broadly neutralizing Abs

100 recombinant Gp120 protein monomers were purchased from Immune Technologies. Purity, protein quality, and testing were performed on recombinant antigens by Immune Technology Corp. Proteins were purified from 293 culture supernatants using nickel columns, and purity was assessed by SDS page by Immune Technology Inc. Additionally, we performed a second SDS-PAGE (Figure B in S1 File) upon receipt of these proteins to ensure that only high-quality, antigens were used for ELISAs and Mass Spectrometry. FPLC was performed on a subset of proteins to spot-check protein quality. New batches of proteins were acquired from Immune Technology if more than 1 band was observed on the SDS-page. Proteins that exhibited more than 1 band across 2 batches were eliminated from consideration, resulting in the final inclusion of only 94 recombinant gp120 monomers in the analysis (S3 Table). PGT121, A32, F105, 447-52D and VRC01 Abs were obtained from the NIH AIDS reagents program. 2G12 and B12 Abs were purchased from Polymun Scientific. PGT122, 123, 124, 125, 126, 127, 128, 135 and 136 were generously provided by Dr Dennis Burton (Scripps Research Institute).

### ELISA-based antigenicity assay

ELISA assays were performed by capturing gp120 monomers on D7324-coated (anti–C-terminal gp120 sheep Ab, Aalto Bioreagents) plates, to directionally and consistently position the recombinant proteins for antibody binding. Nunc Maxisorp 384-well plates were coated overnight at 4°C with 10 μg/ml of D7324 in 0.1M NaHCO3 (pH 8.6). Plates were washed 4 times with PBST (PBS ±0.01% tween) and blocked with PBSA (PBS containing 5% BSA) for 1 hour at room temperature. After 4 washes with PBST gp120 proteins were added at 80 ng/ml final concentration in PBSA (optimal protein concentration for ELISA was evaluated by protein titration assays based on 6 selected proteins from different clades, Figure E in S1 File) and incubated for 2 hours at room temperature. After washing 6 times with PBST, bNAbs were added at 10 μg/ml in assay diluent (PBS containing 5% BSA and 20% sheep serum) and incubated for 2 hours at room temperature. Following 6 washes in PBST, biotin-conjugated mouse anti-human IgG (BD Biosciences) was added to each well at 1:1000 dilution in PBSA and plates were incubated for 1 hour at room temperature. After 6 washes in PBST, high-sensitivity streptavidin-HRP (Pierce) was added to each well at 1:100 dilution in PBSA and incubated for 1 hour at room temperature. Plates were washed 6 times in PBST and developed by adding UltraTMB substrate (Pierce) to each well. Development was stopped by adding 2M sulfuric acid and plates read at OD450 with Tecan 1000 pro reader. Background OD values (wells without gp120) were subtracted from test wells containing gp120 proteins. Two biological replicates and three technical replicates for each bNAb were performed to provide statistical estimation and evaluate assay reproducibility. ELISA data from biological replicates were normalized by median centering to avoid systematic variance, aiding in cross-experiments comparison. Reproducibility between replicate data sets was evaluated by examining the coefficients of variation (CV).

### Identification of sequons within HIV gp120 sequences

94 HIV gp120 sequences were multiply aligned by Clustal Omega (EMBL-EBI), based on the curated alignments HMM model from Los Alamos HIV database [80]. The consensus sequons (N-X-S/T) were identified in each aligned sequence to identify all potential glycosylation sites. The position number of individual sequons for each aligned sequence was mapped back to the HXB2 sequence. Nomenclature for the positions of the sequons not aligned to HXB2 was determined by the previously aligned position present within HXB2; for instance, N137.5 indicates the glycosylation site found at the fifth residue after Position 137 in HXB2.

### Defining glycan occupancy by mass spectrometry

5 μg of each gp120 protein (Immune Technology Corp.) was denatured by incubating with 10 mM of dithiothreitol at 56°C for an hour and alkylated by 55 mM of iodoacetamide for 45 minutes in the dark prior to digestion with proteases (Promega) at 37°C overnight. Various combinations of proteases were used for different samples depending on their particular protein sequences to achieve optimal detection of potential glycosylation sites. The resulting glycopeptides were deglycosylated by incubating with PNGaseF (ProZyme) in the presence of H_2_^18^O water (Cambridge Isotope Laboratories) overnight. The deglycosylated peptides were then dried and reconstituted in 0.1% formic acid. Deamidation of any Asn would result in a mass shift of +1. We purposely controlled for mapping of N-linked sites by performing only the enzymatic deglycosylation in the presence of O18-water so as to generate a mass shift of +3. Following deglycosylation, the sample was immediately removed from O18-water and replaced with non-isotope heavy buffers. We also performed the deglycosylation below pH7 since basic conditions promote deamidation [81]. Finally, when database searching, we allowed for Asn +1 and +3 for all Asn but only observed Asn +3 residues in consensus sequons further suggesting the lack of deamidation. In addition to Asn +1 (deamidation) and Asn +3 (deglycosylation), we also allowed for oxidation of methionine and alkylation of cysteine residues. Peptides were separated on a 75 μm (I.D.) x 15 cm C18 capillary column (packed in house, YMC GEL ODS-AQ120ǺS-5, Waters) and eluted into the nano-electrospray ion source of an Orbitrap Fusion Tribrid mass spectrometer (Thermo Fisher Scientific) with a 180-min linear gradient consisting of 0.5–100% solvent B over 150 min at a flow rate of 200 nL/min. The spray voltage was set to 2.2 kV and the temperature of the heated capillary was set to 280°C. Full MS scans were acquired from m/z 300 to 2000 at 120k resolution, and MS2 scans following collision-induced fragmentation were collected in the ion trap for the most intense ions in the Top-Speed mode within a 3-sec cycle using Fusion instrument software (v1.0, Thermo Fisher Scientific). A protein database was compiled from 94 gp120 protein sequences provided by Immune Technology Corporation. Raw spectra were searched against the gp120 protein database using SEQUEST (Proteome Discoverer 1.4, Thermo Fisher Scientific) with full MS peptide tolerance of 20 ppm and MS2 peptide fragment tolerance of 0.5 Da, and filtered using ProteoIQ (v2.7, Premier Biosoft) at the protein level to generate a 1% false discovery rate for protein assignments. Glycan occupancy *G*_*ai*_ of a potential glycosylation sequon *a*_*i*_ was determined as follows,

$$G_{a_{i}}=\frac{\sum P_{Occup_{{}_{i}}}}{\sum P_{Occup_{{}_{i}}}+\sum P_{Unoccup_{j}}}$$ (1)

G represents a ratio of total spectral counts (generated by ProteoIQ) between the ^18^O-labeled peptides *P*_*Occupi*_ containing the glycosylated sequon *a*_*i*_ and all detected peptides containing the sequon *a*_*i*_ with glycosylated *P*_*Occupi*_ or unglycosylated *P*_*Unoccupi*_ forms. The glycan occupancy threshold for *a*_*i*_ was set as sum of *P*_*Occupi*_ and *P*_*Unoccupi*_ > 10 to avoid instrumental noise at low spectral signal. Occupancy of peptides with multiple sequons was determined by analyzing the ratio of the fragments in MS/MS that in most cases lead to specific quantitative assignment of glycosylated sequons; in a limited set of cases where Asn residues were adjacent or 3 AAs apart and not able to be accurately separated for quantification by multiple unique MS/MS fragments, the sequons were considered to be equally occupied.

### Pairwise covariance analysis between glycan occupancy and antigenicity

To quantitatively assess direct relationships between site-specific glycosylation occupancy and Ab binding, a matrix of values for pairwise Pearson correlations between site occupancy and Ab ELISA binding profiles was constructed. A given correlation coefficient characterizes the strength of the relationship between occupancy levels at individual sites and Ab binding; the sign of the value informs whether glycan presence at that specific site has positive or negative influence on binding.

### Bayesian machine learning algorithm for identifying glycan sites critical for Ab binding

bNAb binding to the HIV envelope usually involves multiple glycans, often located proximal to an epitope. However, distal glycans may also contribute through conformational changes to the envelope. Thus, to define global glycan site combinations concomitantly important for Ab binding, we developed an algorithm that integrated two computational methods. First, a support vector regression (SVR) algorithm that implements support vector machines (SVMs) [69] was employed as a supervised learning model to predict Ab binding activities based on glycan site occupancy values. Second, a Bayesian Markov Chain Monte Carlo (MCMC) with the biased random walk Metropolis–Hastings algorithm [70, 71] was employed to approximate the high-dimensional posterior distribution within the data to identify the optimal combinatorial glycan determinants that best fit the model predictions for binding with the experimental data.

*The model*. We identified 110 sequons from the alignment of the 94 gp120 sequences utilized for the Ab binding profiles. We hypothesized that each sequon could be classified as either a “determinant” (critical for binding) or a “non-determinant” (not critical for binding) for Ab binding. A determinant could either be an agonist or antagonist. The calculation to identify glycan determinants was based on a joint probability distribution *P*(*X*) from a multi-dimensional sequon space:

$$\begin{array}{l} \mathrm{Joint}\;\mathrm{probability}=P(X),\\ X=\{x_{1},x_{2},.....x_{110}\}\\ x_{i}=\left\{ \begin{array}{ll} 1 & \mathrm{determinant}\\ 0 & \mathrm{otherwise} \end{array}\right. \end{array}$$ (2)

where the sequon index *x*_*i*_ indicates a dimension, and contains two possible states [0, 1]. The complete probability distribution containing 2^110^ states is overwhelming to exhaustive sampling. Therefore, we used an approximation to provide a statistical estimate of the glycan determinant distribution *P(X)* given a set of experimental observations. According to Bayes’ theorem, the conditional probabilities of the glycan determinants given an observed data set are:

$$p(X_{opt}|data)=\frac{p(data|X_{opt})p(X_{opt})}{p(data)}$$ (3)

where *X*_*opt*_ denotes the vector of the glycan determinants in the model and *p*(*X*_*opt*_ | *data*) represents the posterior of *X*_*opt*_. *p*(*data | X*_*opt*_) and *p*(*X*_*opt*_) are likelihood and prior of *X*_*opt*_, respectively. Since the ratio of posterior is only required following MCMC sampling, the equation simplifies:

$$p(X_{opt}|data)\propto p(data|X_{opt})p(X_{opt})$$ (4)

Taking a log transformation then yields the sum of the log likelihood and the log prior:

$$\begin{array}{l} -\ln(posterior(X_{opt}))=\\ -\ln(likelihood(X_{opt}))-\ln(prior(X_{opt})) \end{array}$$ (5)

*–ln*(*likelihood*(*X*_*opt*_)) was calculated by mean squared errors (MSE) between the prediction from SVR and the observed data. SVR was trained on a sub-set of glycan site occupancy profiles (*X*_*opt*_), and examined by means of 10-fold cross-validation randomly dividing the data into training and test sets with fitness estimated by calculating MSE. -ln(*prior*(*X*_*opt*_)) was calculated by the sum of squared deviation between the selected and empirical glycan determinants, assuming that the distribution of the site identified between two states [0, 1] follows a Gaussian distribution:

$$-ln(prior(X_{opt}))=\sum_{j}\frac{1}{2\sigma_{j}^{2}}{\left(x_{j}-\langle x_{j}\rangle \right)}^{2}$$ (6)

*σ*^*2*^_*j*_ and <*x*_*j*_> are variance and mean of glycan occupancy at the site *x*_*j*_. To sample the desired probability distribution *P(X)* we implemented a Metropolis–Hastings MCMC walk, in which the algorithm iteratively computed the posterior at current and next position. With this posterior assumed proportional to *P(X)*, the decision of jumping to the next position was then obtained:

$$\begin{array}{l} X_{s+1}=\left\{ \begin{array}{l} X'\;\mathrm{if}\;Unif\;\left(0,1\right)\leq \alpha \left(X_{s},X'\right)\\ X_{s}\;\mathrm{otherwise} \end{array}\right.\\ \alpha \left(X_{s},X'\right)=\min\left(1,\frac{posterior\left(X'\right)}{posterior\left(X_{s}\right)}\right) \end{array}$$ (7)

*X*_*s*_ indicates the current position and *X’* the candidate for the next position; *X*_*s+1*_ the next position. The distribution *α* of the next position depends only on the current position value, by definition of the Markov chain. The algorithm accordingly generated a sequence of sample values in which the distribution of values closely approximated the original probability distribution *P*(*X*).

The calculation procedure started from a randomly selected set of glycan determinants and performed 50,000 iterations of MCMC walk for each Ab, in order to converge on a desired distribution (Figure M in S1 File); the first 5,000 iterations were discarded as a burn-in period which identified very different distributions. The output of this calculation provided marginal probabilities for each sequon identified as a determinant (Figure N in S1 File). We performed 10 cycles of MCMC walk for each Ab to avoid the potential problem of the search getting trapped in local optima. To estimate statistical significance among the sequons, a background model was calculated in which the original glycan occupancy matrix was permuted by shuffling the order of gp120 proteins to preserve the heterogeneity of glycan occupancy yet capture noise effects. This background model was performed for each Ab using 100 permutations to estimate null distribution for individual sequons, with the null hypothesis posed that the glycan at the site has no impact on the Ab binding. Only the sites whose *q*-value after multiple-testing adjustment showed statistical significance (*q*<0.01) (Figure F in S1 File), rejecting the null hypothesis, were identified as glycan site “determinants”. We used a customized Matlab script for Metropolis–Hastings MCMC algorithm, implemented Matlab LIBSVM package [82] for SVR with SVR parameters, kernel type = linear, cost = 0.75, and the epsilon = 0.1. The algorithm was run on Linux computing clusters.

To evaluate whether model balance between under- or over-fitting, a learning curve technique [83] was employed to calculate training error and cross-validation error as a function of training set size.

$$\begin{array}{l} E_{train}(\theta )=\frac{1}{2N}\sum_{i=1}^{N}{\left(J_{\theta }(x^{\left(i\right)})-y^{\left(i\right)}\right)}^{2}\\ E_{cv}(\theta )=\frac{1}{2N_{cv}}\sum_{i=1}^{N_{cv}}{\left(J_{\theta }(x_{cv}^{\left(i\right)})-y_{cv}^{\left(i\right)}\right)}^{2} \end{array}$$ (8)

where *N* indicates the training set size and *N*_*cv*_ the testing set size in cross-validation. *J*_*θ*_(*x*) denotes the regression function of SVR and *y* represents the actual Ab binding fingerprint. A learning curve was used to evaluate the model fitting given the predictors (Figure O in S1 File). An under-fitting model was recognized if only one glycan site occupancy profile was found as a predictor, in which large training errors quickly emerged for a small training set (Figure O in S1 File, the left panel). An over-fitting model was obtained if all glycan occupancy profiles were found as predictors, in which the cross-validation errors decreased shortly but then grew as the training set size increased further (Figure O in S1 File, the middle panel). A model using the glycan site determinants identified from the Bayesian machine learning algorithm as the predictors showed excellent fitting behavior, in which both the training and cross-validation errors converged at the level of the expected errors from experimental noise (Figure O in S1 File, the right panel).

To determine how the glycan determinants modulate Ab recognition, the directional weight (*DW*) was introduced and calculated:

$$\begin{array}{l} DW=p(x_{i})\times d\\ d=\left\{ \begin{array}{ll} 1 & \mathrm{if}\;\mathrm{corr}({\mathrm{Ab}}_{y},G_{x_{i}})>0\\ ‑1 & \mathrm{otherwise} \end{array}\right. \end{array}$$ (9)

*p*(*x*_*i*_) denotes the probability of a sequon being a “determinant” which was obtained from the MCMC-SVR model and *d* represents a direction [1, –1] determined by the covariance matrix between individual glycan occupancy pattern versus the Ab fingerprint (Fig 2C).

### Identifying amino acid positions critical for Ab binding by Bayesian MCMC-SVR

Bayesian MCMC-SVR was then extended to identify protein residues critical for Ab binding. The peptide sequences of 94 gp120 proteins were pre-processed to remove less informative positions. Based on multiple sequence alignment, 534 aligned positions were identified across the 94 proteins. Positions at which the residues were identical in all proteins or where more than 10% of the proteins had unknown residues, denoted as gaps, were removed, leaving only 303 common positions. For importing these data into the model, the finalized sequence data was encoded as a 6,363–dimensional vector (303 positions × 21 amino acid types including the gap), where every single residue of the 303 positions in each sequence was converted from a categorized amino acid into a binary vector. Ultimately, three models—glycan alone, sequence alone, and both glycan and sequence—were constructed for each Ab to assess the model prediction performance as well as identify the critical glycan site and protein residues that may tune Ab binding.

### *De Novo* computational design for antigenically optimized antigens via glycan-engineering

Using the Bayesian MCMC-SVR algorithm that identified the glycan determinants positively or negatively impacting Ab binding, the data were further analyzed to rationally design antigens with selective Ab antigenic-profiles. The same training set (glycan occupancy, Ab fingerprints and envelope sequences) and model algorithm were utilized, but now modified to search for global optimum solutions for best or worst binding (the objective function *-ln*(*posterior*(*X*_*opt*_)) is modified). Given a set of previously identified Ab-specific glycan determinant sites denoted as *X*, the model in this section attempted to determine the likelihood of the presence or absence of each critical site associated with the optimized Ab binding:

$$\begin{array}{l} \mathrm{Joint}\;\mathrm{probability}\;\mathrm{of}\;\mathrm{the}\;\mathrm{optimized}\;\mathrm{binding}=P(X)\\ X=\{x_{1},x_{2},.....x_{m}\}\\ x_{i}=\left\{ \begin{array}{ll} 1 & \mathrm{Presence}\\ 0 & \mathrm{Absence} \end{array}\right. \end{array}$$ (10)

where each glycan determinant *x*_*i*_ contained two states [0, 1]. Thus, the model aimed to approximate the joint probability distribution *P(X)* by implementing the same Bayesian MCMC sampling as earlier (Eq 5).–*ln*(*posterior*(*X*_*opt*_)) represented the predicted antigenicity from the trained SVR model given a set of combinatorial sites *X*_*opt*_.

For sampling within the multi-dimensional probability distribution *P*(*X*), we performed 200,000 iterations to reach convergence from an initial gp120 sequence, and estimated the global sequon occupancy profile for an antigen that yields optimized Ab binding. Each iteration simulated the evolutionary process of mutagenesis, in which the state of a selected site *x*_*i*_ was altered between 0 and 1 analogous to random knockout/insertion of the sequon; an evolutionary driving force is assumed to mainly depend on predicted antigenicity. Accordingly, in each iteration determination was made of whether the sequon should be added or removed by computing the ratio of posteriors for the current and previous iteration according to Eq 7; that is, we computed the ratio of predicted antigenicity between current versus previous iterations. The final output of the model was the marginal probabilities of two states at every glycan determinant. High probability of state 1 suggested that the glycan determinant most likely facilitated Ab binding, so that the sequon should be included in the optimal antigen design. Conversely, high probability of state 0 suggested that occupancy at that sequon would impair Ab recognition, suggesting that this site should be excluded. Ultimately, the model was able to identify optimized profiles of glycan determinants on the antigens with ideal antigenicity.

When generating a *de novo* peptide sequence from a gp120 sequence template to accommodate an ideal glycan determinant profile, addition and deletion of sequons was accomplished according to these rules: (1) if a sequon needed to be eliminated, a Asn-to-Gln substitution at the sequon was introduced; (2) if a new sequon needed to be introduced, a N-L-T sequence was substituted into the original sequence (although if multiple sequons were modeled that were in close proximity [e.g., N187.6 and N187.7] only the first site would be added to avoid steric hindrance).

### Incorporating steric effects of the glycans into gp120 antigen design

To incorporate the steric hindrance effects of proximal glycans, pairs that negatively impacted one another were identified. Our hypothesis was that if two glycans sterically constrained each other, the glycan occupancy patterns should be mutually exclusive; i.e., they should have strong negative correlation. Therefore, a covariance matrix was constructed containing all pairs of glycan-glycan auto-correlations. To determine statistical significance, a nominal P-value of each pair was calculated, assuming that the distribution of all pairs of correlation coefficients followed a Gaussian process, and an adjusted *p*-value was corrected to account for multiple hypothesis testing (False discovery rate adjusted *p*-value < 0.1). Glycan pairs with significant mutual exclusion were then identified (Figure J in S1 File). Next, to estimate the total degree of steric hindrance potentially induced on the epitope of the antigen, the mutually exclusive glycan pairs were taken into account. Each glycan pair possessed a steric hindrance weight based on the glycan-glycan correlation coefficient, and total weights of the included glycan pairs were summed up to represent total degree of steric hindrance. Mean and s.d. of the steric hindrance were estimated across all 94 gp120 proteins which represented the basal level of the steric hindrance occurring on naïve gp120 proteins. To incorporate the steric hindrance effects on antigen design, the antigens, after *de novo* design, were then evaluated for total degree of steric hindrance. Steric hindrance levels greater than the basal level (> mean + 1 s.d.) were considered to be larger than acceptable. Glycan sites that showed the least effect on bNAb binding from steric hindrance were removed one at a time until an acceptable level of steric hindrance was achieved. The process of removing steric glycan sites was repeated until total degree of steric hindrance was lower than mean+1 s.d.

### Recombinant Env glycoprotein expression and purification

The sequence encoding wild-type MF535.W0M.ENV.D11 gp120 and PGT121^+/-^/PGT128^+/-^ optimized glycoproteins, including a signal peptide-‘MPMGSLQPLATLYLLGMLVASVLA’ at N-terminus and an avitag-‘GLNDIFEAQKIEWHE’ followed by a histag-'HHHHHH’ at the C-terminus, were synthesized and inserted into pcDNA3.1 (Thermo Fisher). Plasmid DNA was purified and verified by sequencing. Plasmids encoding these proteins were transfected into 293-F cells (Life Technologies, cat. no. R790-07) and proteins were isolated from expression supernatants 6 days after transfection. Briefly, gp120 proteins were purified by metal affinity chromatography using Ni-NTA resin (Qiagen). Fractions containing gp120 were combined and oligomers, trimers and monomers were separated by gel filtration chromatography using a Hi-Load 16/60 Superdex 200pg column (GE Healthcare). Protein purity was confirmed by SDS-PAGE gel electrophoresis and Western blotting using a mouse monoclonal Ab Chessie 13–39.1 (NIH, AIDS Reagents Program) (Figure H in S1 File).

### Code availability

MATLAB source codes for the Bayesian MCMC-SVR algorithm to identify glycan/sequence determinants, and the *de novo* antigen optimization design program, can be accessed from Supplementary Information.

## Supporting information

S1 File Supplemental tables and figures. (DOCX)

S2 File The sequences of WT gp120 (MF535.W0M.ENV.D11) and the glyco-engineered proteins tested in this study. (PDF)

S3 File The codes for Bayesian MCMC-SVR algorithm and *de novo* antigen optimization design. (ZIP)

S1 Table List of recombinant gp120 proteins. (PDF)

S2 Table Site-specific glycan occupancy across gp120 proteins. (XLSX)

S3 Table Model-identified antibody glycan site determinants and critical protein residue positions. (XLSX)
