# Supplementary material for: Exploiting glycan topography for computational design of Env glycoprotein antigenicity
Source: PLoS Comput Biol. 2018 Apr 20;14(4):e1006093. doi: 10.1371/journal.pcbi.1006093 (PMC5931682; doi:10.1371/journal.pcbi.1006093)
Supplement: S1 File — (DOCX) [file pcbi.1006093.s001.docx]

# Supplemental information

## Table A

## Evaluation of glycan occupancy by mass spectrometry.

| Gp120 ID/Name | Sequon site | Peptide | Site substitution  (N->D) | Peptide mix ratio | Spectral count ratio |
| --- | --- | --- | --- | --- | --- |
| 8/MI206.W0M.ENV.D1 | N197 | LINC**N**TSAITQACPK | LINC**D**TSAITQACPK | 1:1 | 1.02 |
| 28/ZM109F.PB4 | N276 | IVIRSE**N**LTDNAK | IVIRSE**D**LTDNAK | 1:1 | 0.94 |
| 37/MN | N295 | SVQI**N**CTRPNYNKR | SVQI**D**CTRPNYNKR | 1:1 | 0.96 |
| 28/ZM109F.PB4 | N160 | HCSF**N**ITTDVK | HCSF**D**ITTDVK | 1:1 | 1.02 |
| 28/ZM109F.PB4 | N160 | HCSF**N**ITTDVK | HCSF**D**ITTDVK | 1:5 | 0.2 |
| 28/ZM109F.PB4 | N160 | HCSF**N**ITTDVK | HCSF**D**ITTDVK | 5:1 | 4.61 |

## Table B

## Summary of the glycoproteome analysis across gp120 proteins.

| Total #Samples | Glycosylation site detection rate | Potential sites | Detected sites | Fully occupied sites  (100 %) | Partially occupied sites | Unoccupied sites | Undetected sites |
| --- | --- | --- | --- | --- | --- | --- | --- |
| 94 | 83.4% | 2476 | 2066 | 460 | 1442 | 164 | 410 |

**
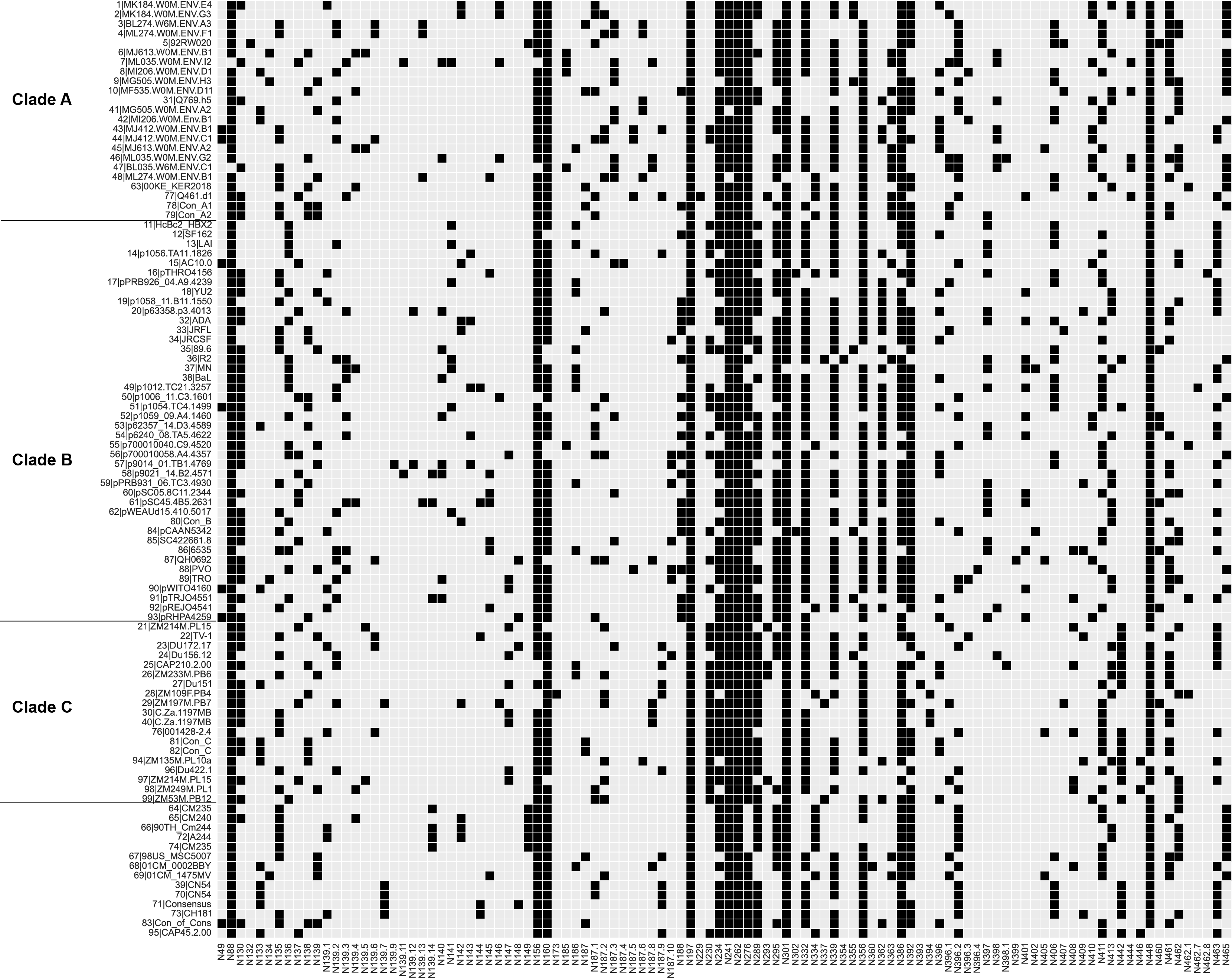
****Figure A**

**Genetic heterogeneity of global N-linked glycosylation sites across our 94 gp120 protein panel.** N-linked glycosylation sequon site presence (x-axis) for each gp120 protein (y-axis) is depicted in the color grid. The absence of a sequon is depicted in a grey. The presence of a sequon is depicted in black (N-X-S/T, X = all amino acids except of P). The positions of the non-canonical N-glycan sites, not aligned to HBX2 sequence, were shown in decimal number, where integral and fractional part indicate the position of the previously aligned protein residue and the number of the residues following the aligned residue, respectively.

**
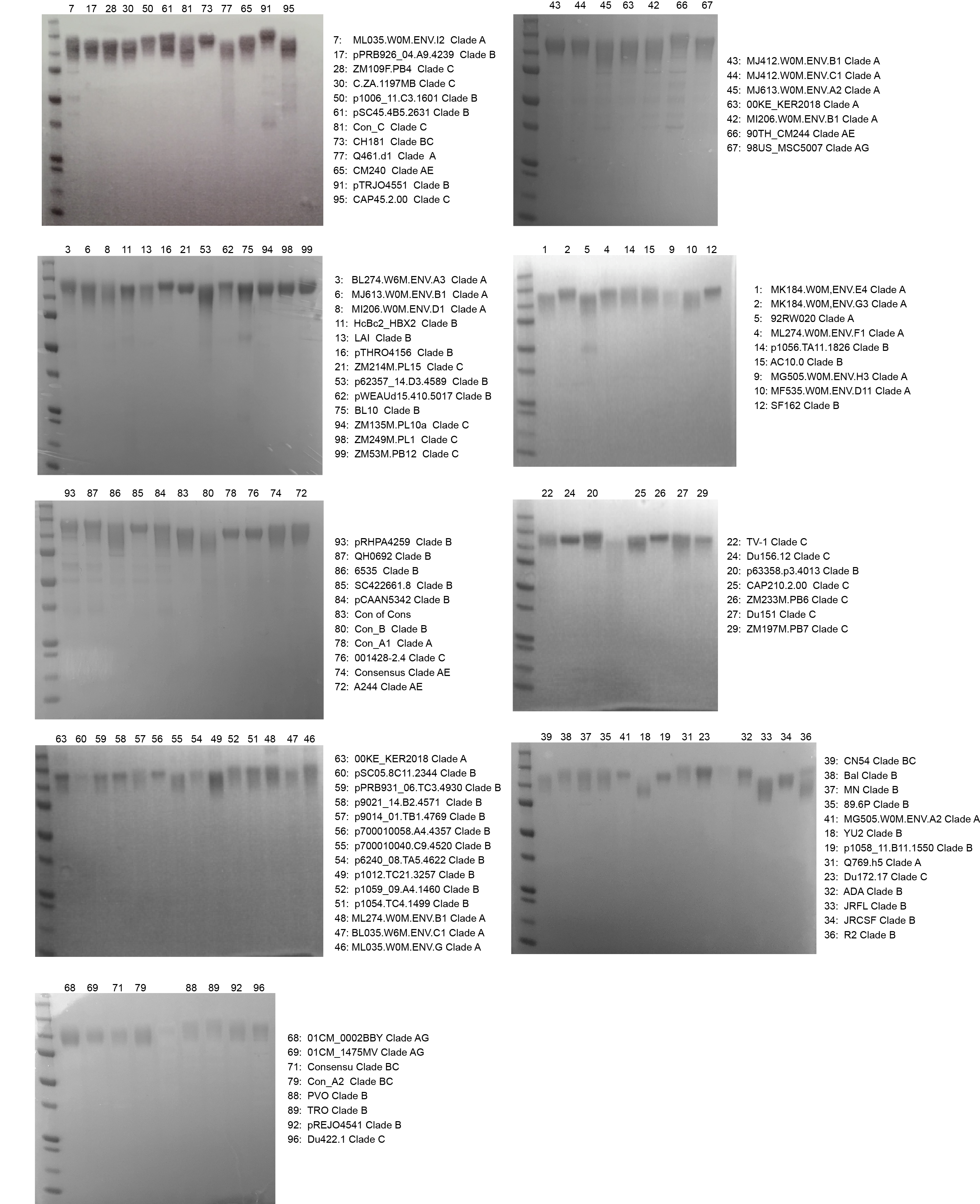
**

**Figure B**

**Protein purity of the gp120 proteins.** The 94 gp120 protein panels purchased from Immune Technologies were tested the purity by SDS-PAGE gel electrophoresis. Further purification was performed if needed. Proteins that showed less than 80% purity were eliminated.


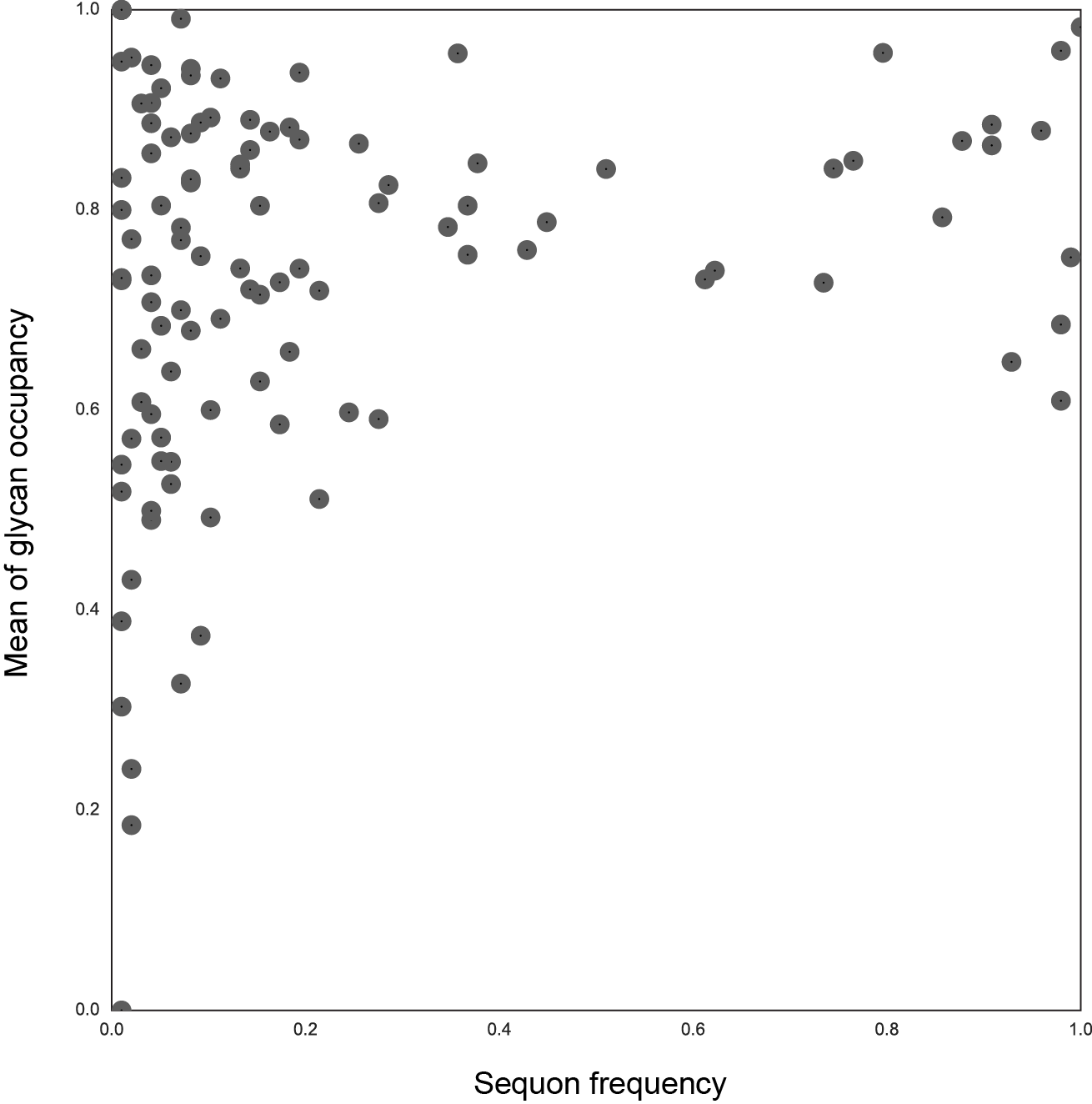


**Figure C**

**The relationship of sequon frequency and glycan occupancy**. The correlation plot depicts the relationship between the frequency of consensus sequons present at each potential N-glycan site and the mean glycan occupancy at each site across all strains.

**
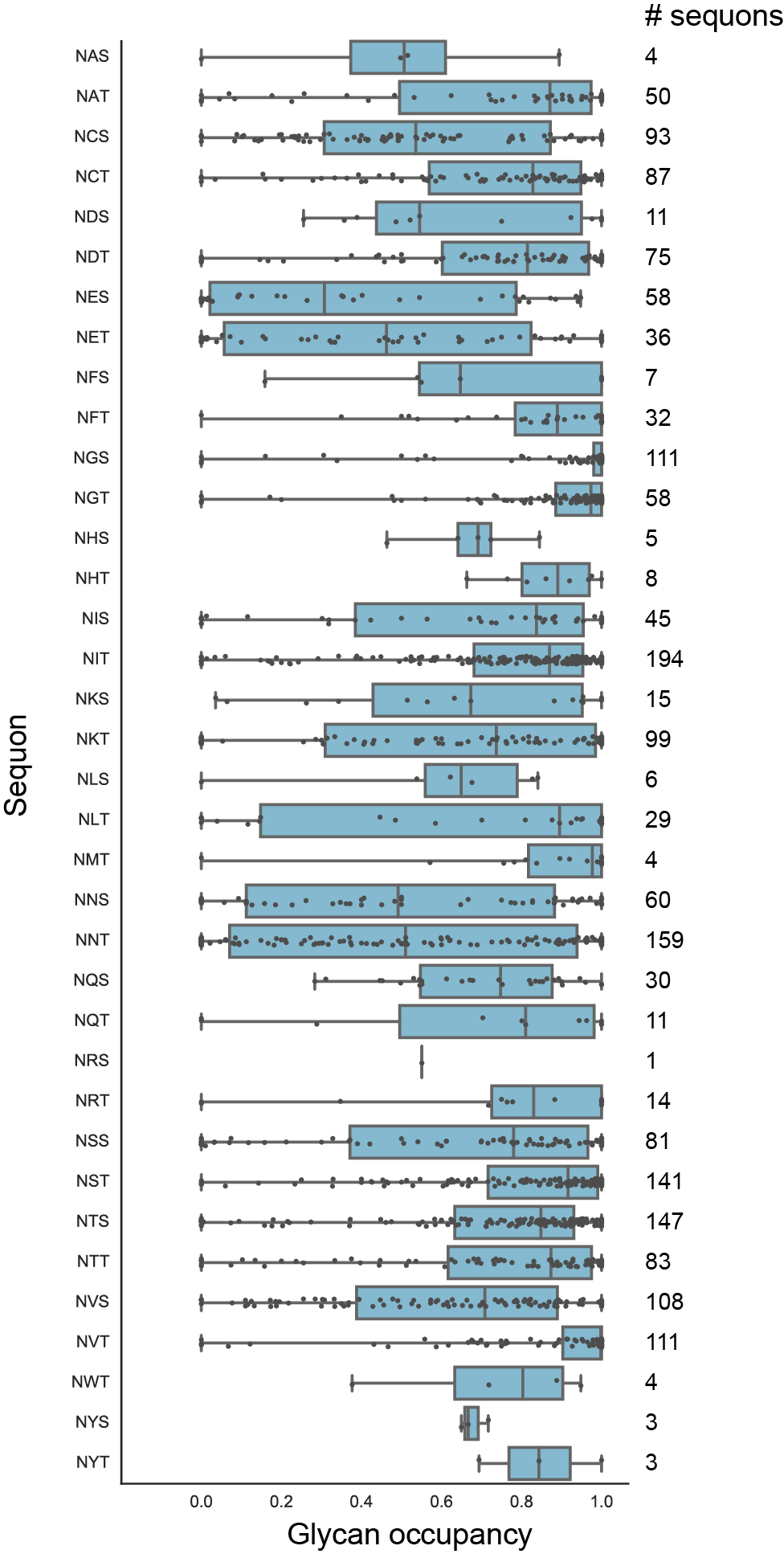
**

**Figure D**

**Variation in glycan occupancy and sequon usage across different sequons.** The box plots show the distribution of glycan occupancy levels (x-axis) at each sequons (N-X-S/T) (y-axis) across all 94 gp120 proteins. The number of distinct sequon sites found in 94 proteins was in the right side.

**
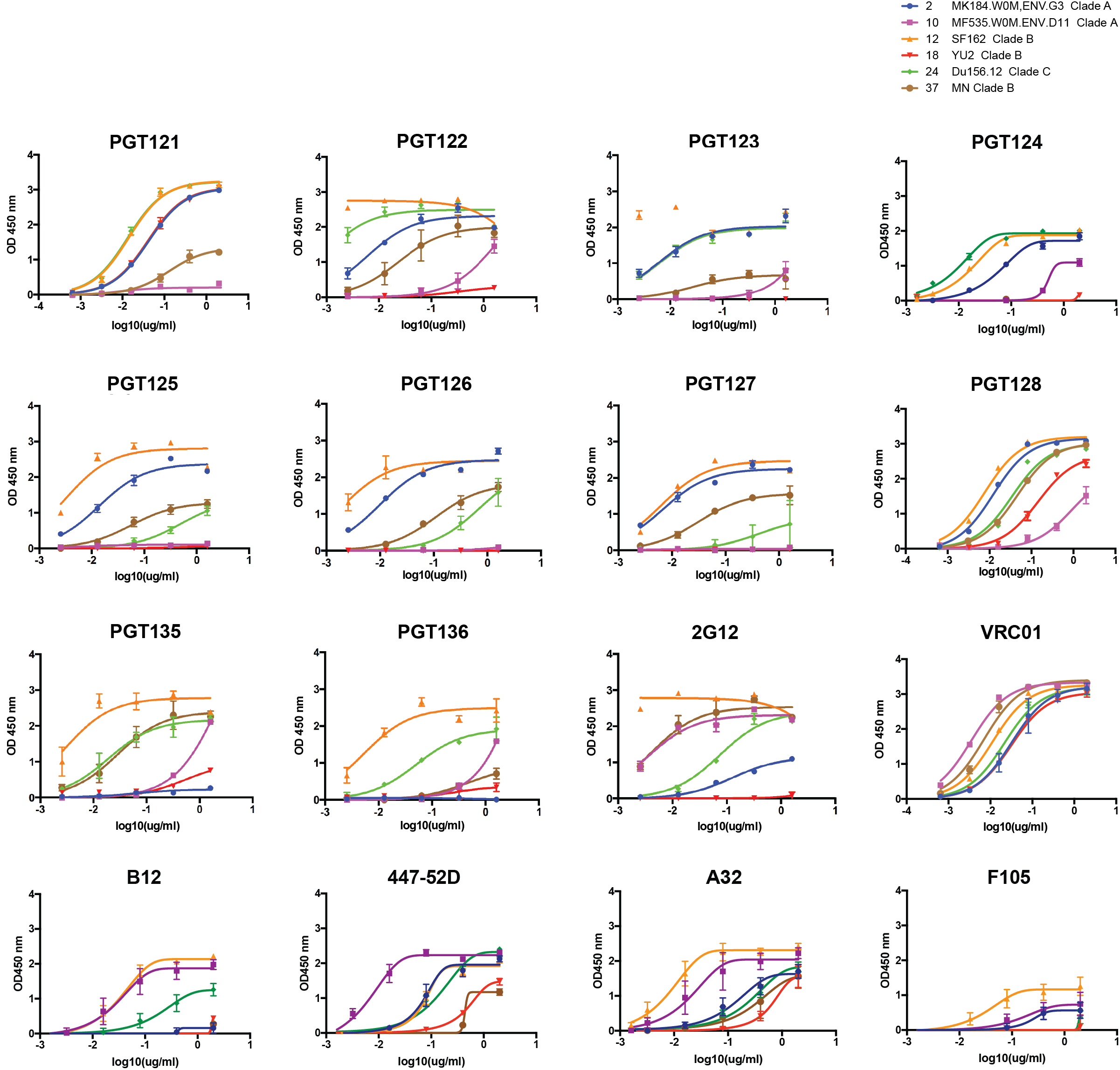
Figure E**

**Binding antibody titration curves.** 6he binding profile for each antibody were measured by ELISA in a dilution series. Six proteins are depicted, representing different clades. Error bars indicate the standard deviation (SD) from total six replicates.

1. (b)


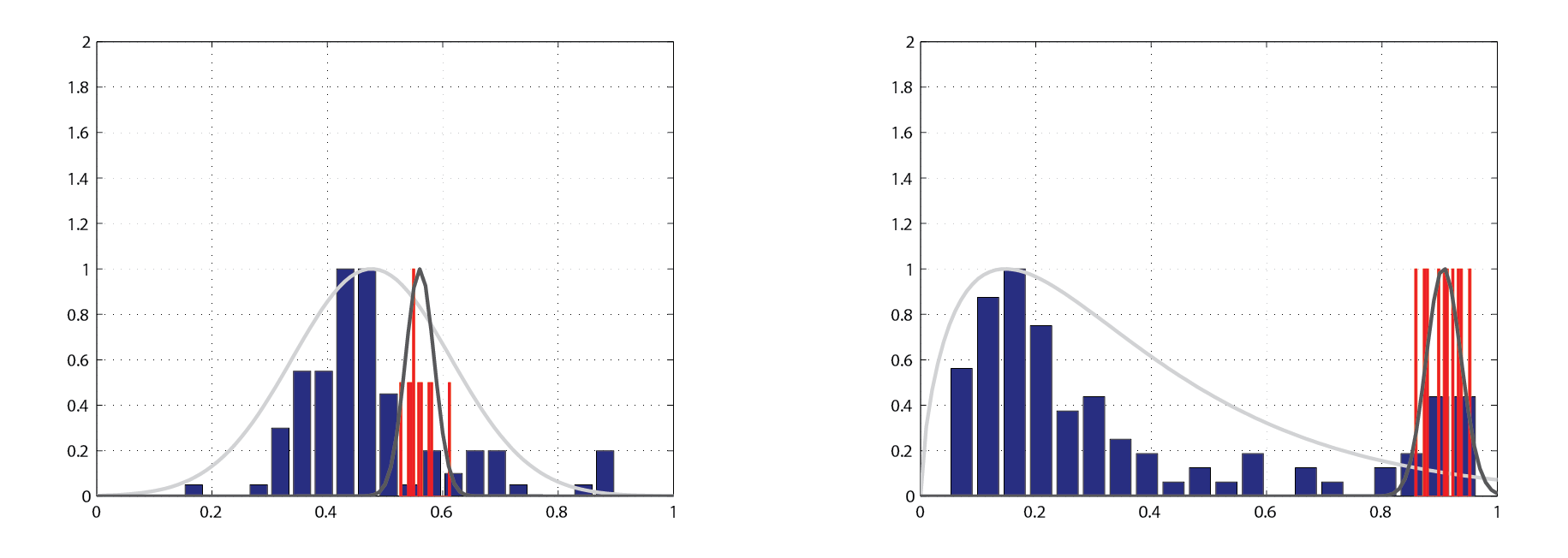


*p* = 1e-7

N322

*p* = 0.355

N140

## Figure F

**Example of the method used to define whether a glycan is significantly important for a specific antibody binding profile.** Glycan occupancy at N140 (left) and at N332 (right) are illustrated as examples of the methodology utilized to assess the importance of glycan occupancy at specific sequon sites on shaping PGT121 binding. Within the Bayesian machine learning model, a nominal *p­­*-value is calculated for each glycan by comparing the marginal probability distribution (the probabilities of that sequon playing a critical role in shaping antibody binding from 10 repeated cycles of Bayesian MCMC walks) of each individual glycan as red bars to a background null distribution (which was estimated by 100 cycles of permutations as blue bars. The smooth curves represent Gaussian distribution fits for the histograms.

##
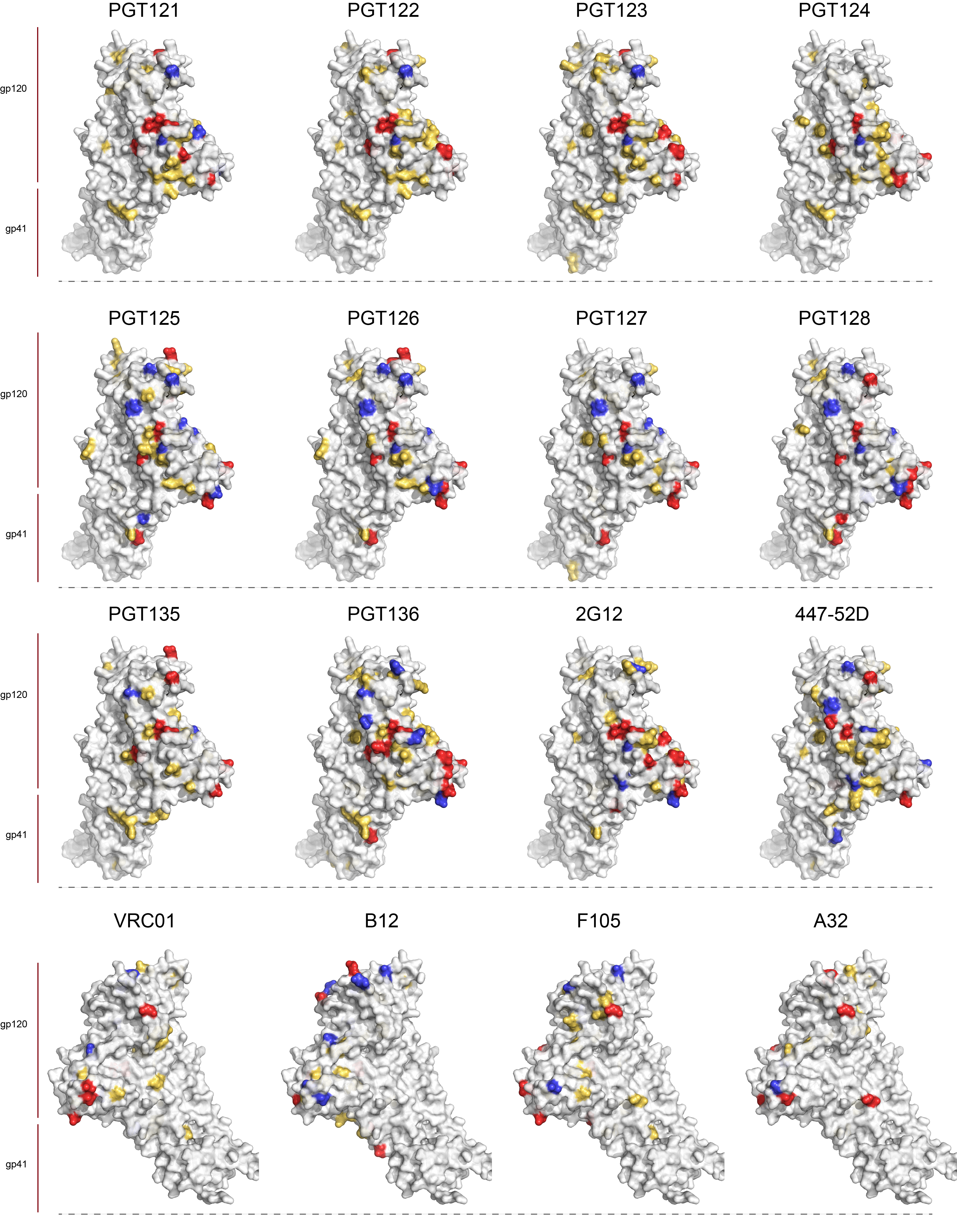
Figure G

**3D rendering of critical glycan and protein residue positions on gp120.** The glycan determinants identified from the Bayesian machine learning model, as shown in Fig. 3d, are mapped here on the gp120 monomer. Agonistic and antagonistic glycans were colored by red (agonist) and blue (antagonist), respectively, and the protein residues are shown in yellow.


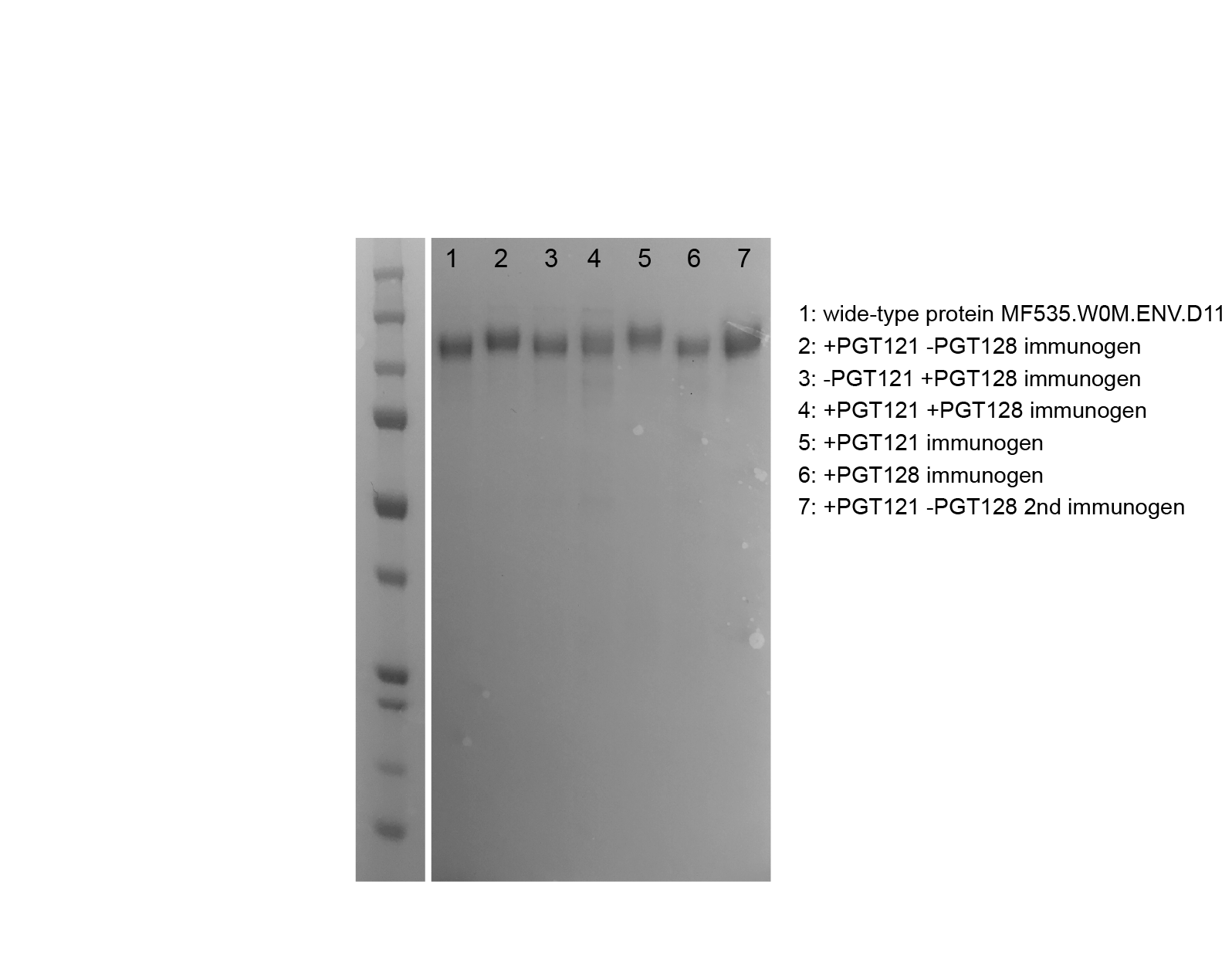


**Figure H**

**Protein purity of WT and the 6 glycoengineered gp120 proteins.** All wide-type and glycoengineered gp120 proteins were expressed in 293T cells and purified using 6xHistidine-tag. The gp120 monomers were isolated by the gel filtration column using with FPLC system.

**
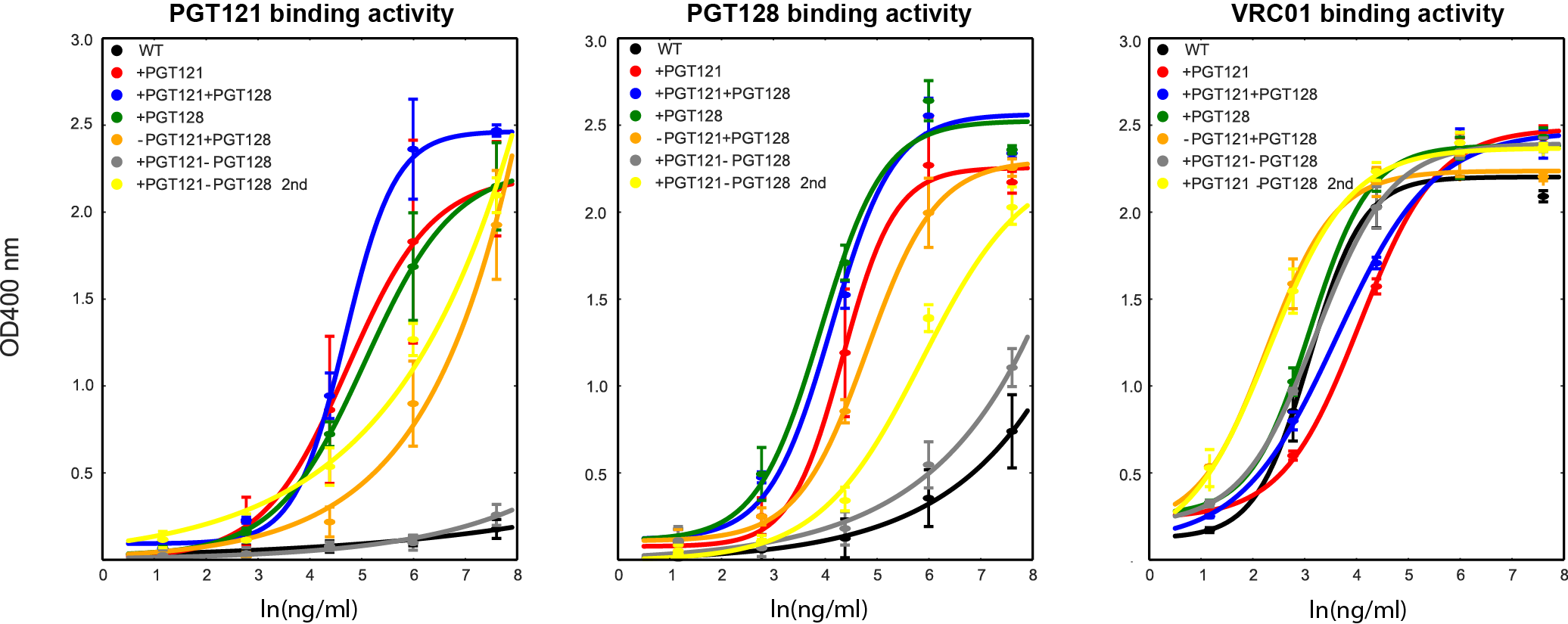
Figure I**

**Antigen-antibody titration curves**. PGT121, PGT128 and VRC01 binding was measured by ELISA in a series dilution of the WT protein (MF535.W0M.ENV.D11 clade A) and 6 new designed antigens. Error bars indicate the standard deviation (SD) from total six replicates.

**Figure J**

**Mapping steric interaction effects among glycans.** Glycan interference connection, in terms of potential steric effects between glycan pairs, are illustrated as networks. Nodes represent individual sequons, and edges indicate that the adjacent nodes have statistically significant mutually exclusive relationships based on the respective two-glycan occupancy patterns. The width of an edge characterizes the degree of steric hindrance between the two glycans, which was calculated using a Pearson correlation coefficient.

**
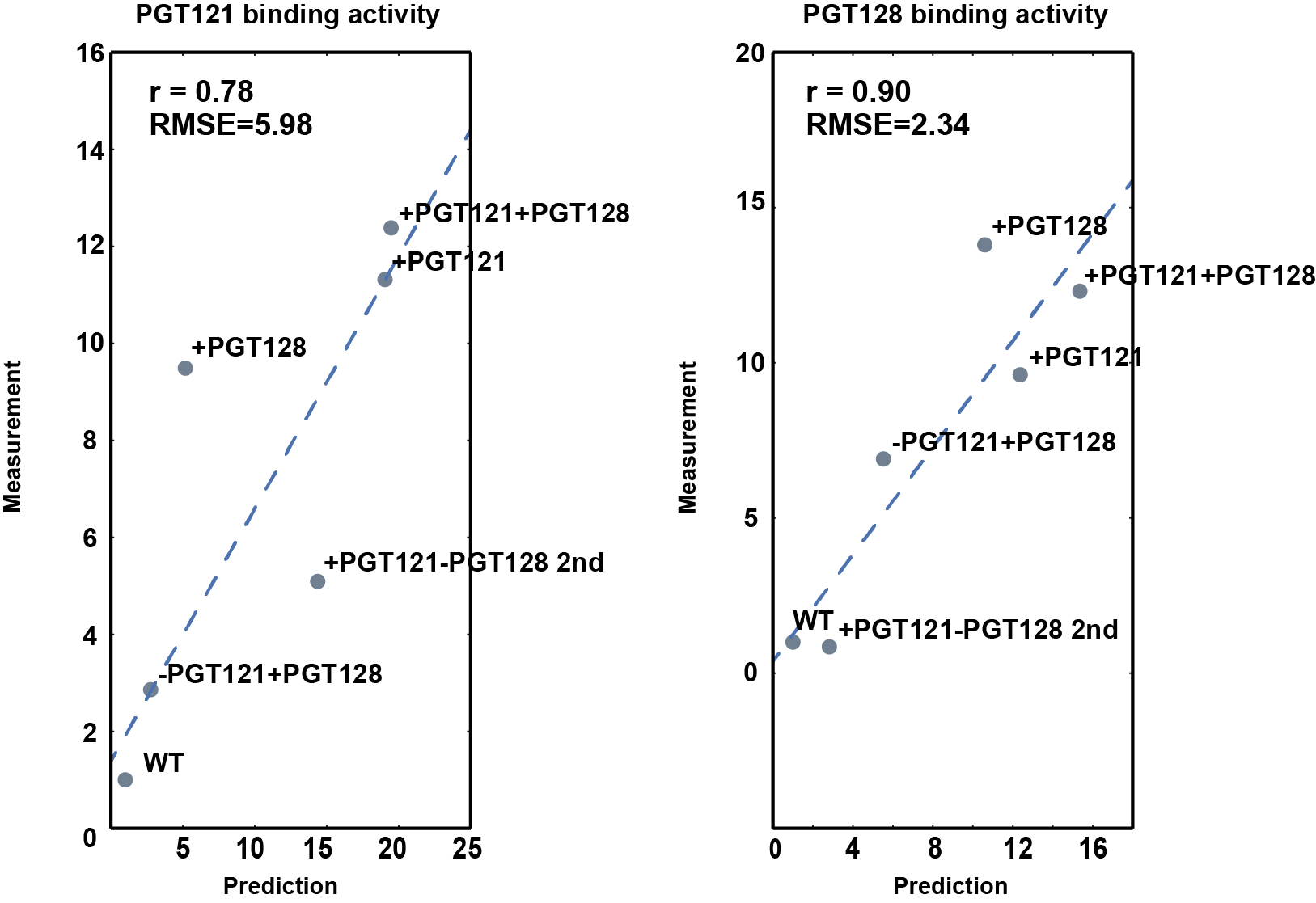
**

**Figure K**

**Benchmarking *de novo* immunogen optimization design.** A goodness-of-fit between model prediction (x-axis) and ELISA readout (y-axis) was measured by correlation coefficient and root-mean-squared-error (RMSE). The binding activities of PGT121 and PGT128 against WT protein and 5 glycanengineered immunogens are shown in Fig. 4c.

**
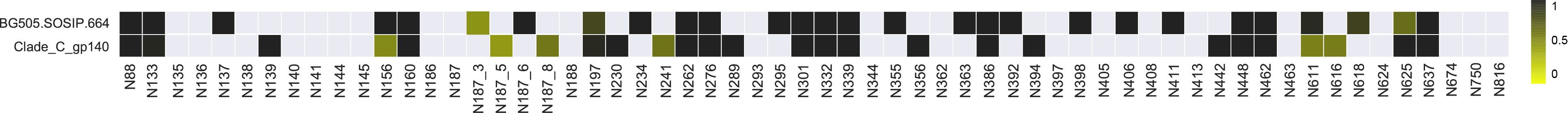
a**

| Total #Samples | Glycosylation site detection rate | Potential sites | Detected sites | Fully occupied sites  (100 %) | Partially occupied sites | Unoccupied sites | Undetected sites |
| --- | --- | --- | --- | --- | --- | --- | --- |
| 2 | 92.5% | 54 | 50 | 36 | 14 | 0 | 4 |

**b**

**c**

**Figure L**

**The glycoproteomic analysis of** **Env** **trimers.** Site-specific glycan occupancy of two gp140 trimers- BG505 SOSIP.664 and Clade C CZA97.012 were analyzed by mass spectrometry. (a) The site utilizations are depicted using a yellow-to-black gradient at each potential glycosylation site. (b) The overall protein coverage. (c) A pairwise comparison of site-specific glycan occupancy of BG505 SOSIP.664 generated in house and from Cao et al., 2017.

##
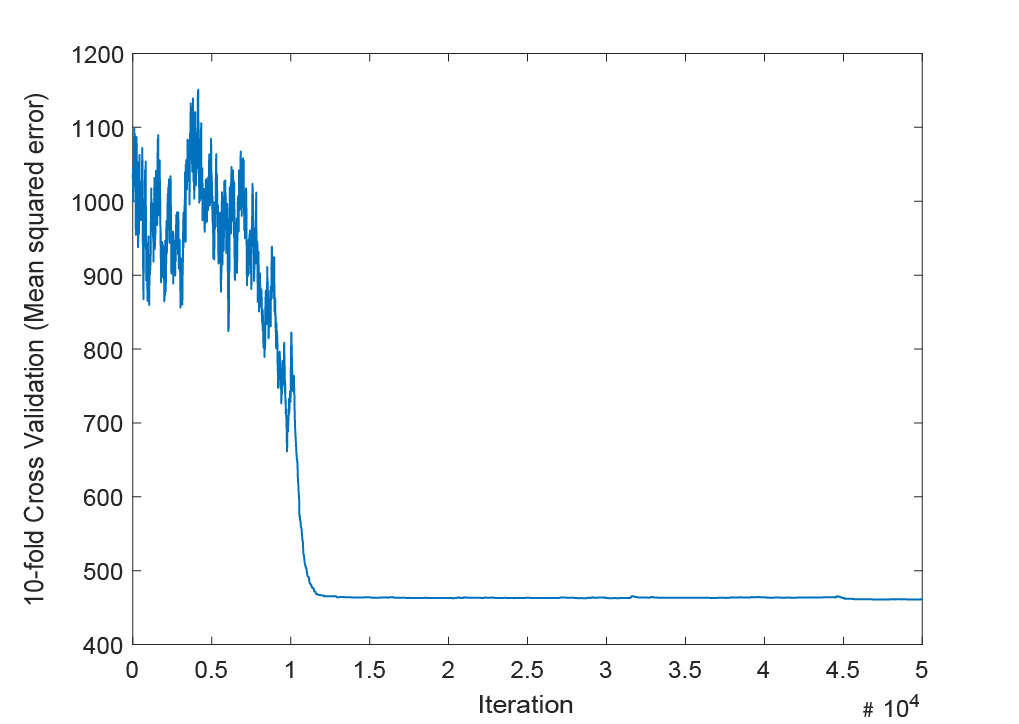
Figure M

**MCMC sampling to optimize model prediction**. A MCMC walk was performed to randomly select a set of combinatorial features (sequon or sequence) for model training, and model prediction was evaluated by calculating mean squared error (y-axis) using a 10-fold cross-validation. The process was repeated 50,000 times (x-axis) to assess the convergence of the model prediction.


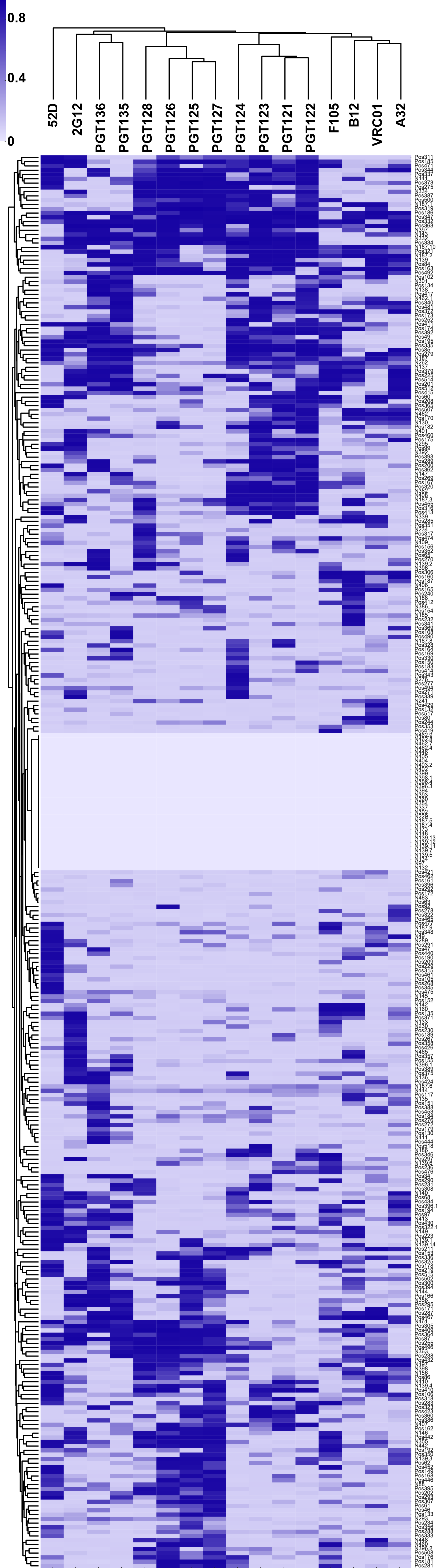


##

## Figure N

**Glycan site and protein residue importance for antibody binding**. The importance of glycan site occupancy or protein residue at any given sequon (x-axis) for each antibody binding profile (y-axis) is depicted in the heatmap. Binding profile similarities are indicated along the top x-axis, based on unsupervised clustering. The marginal probability of importance of individual glycans and sequence position is designated by the intensity of the purple color, according to the legend in the top left corner.

**s**

##
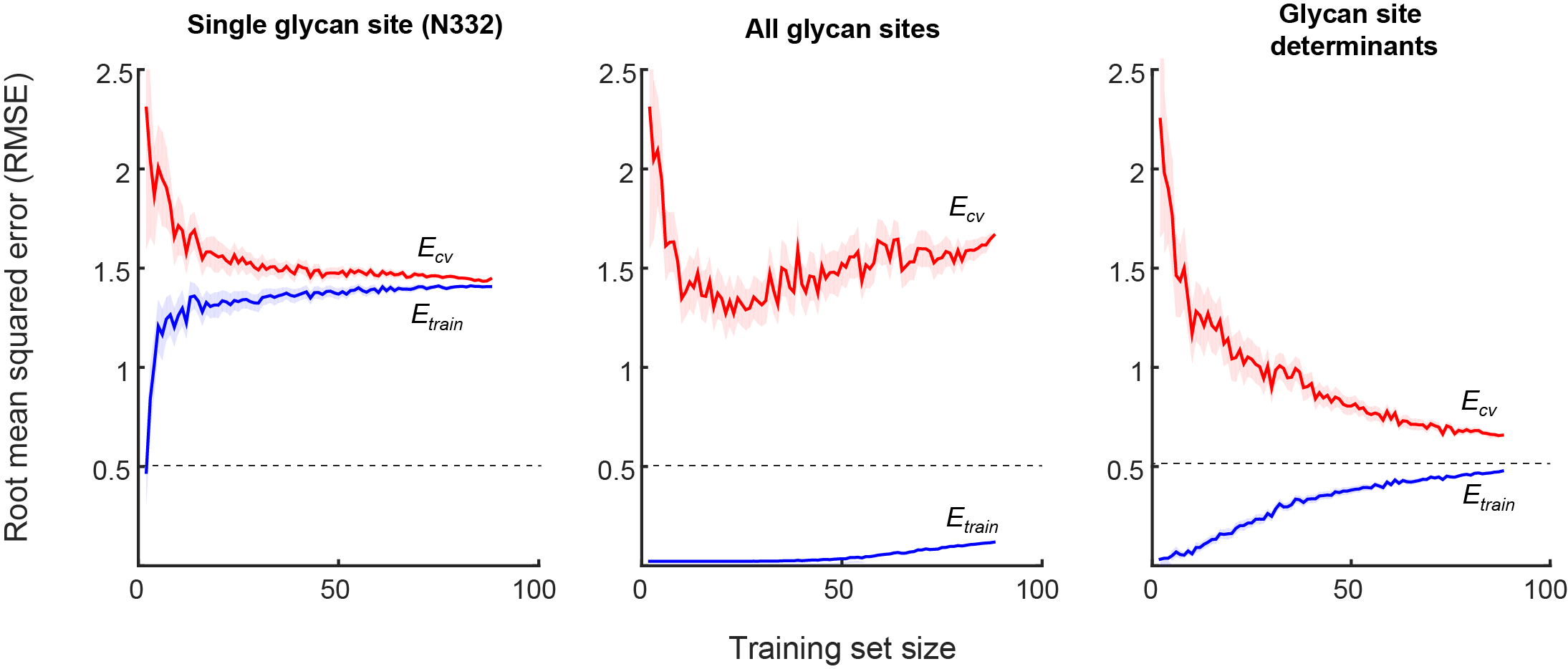
Figure O

**The evaluation of MCMC-SVR model prediction using learning curves.** To evaluate whether the model fully captured the biological variation in the data, without over-fitting, the learning curves were examined by calculating the training error (*E_train_*, the blue line) and the cross-validation error (*E_cv_*, the red line) as a function of the number of training samples (x-axis). The lines represent the average of bootstrapping with 100 times and the shaded area indicated the 95 % confidence interval. In the first test (the left panel), the support vector regression (SVR) was trained only by one of glycan site occupancy profile (N332 in this case) and predicted the bNAb binding fingerprint (PGT121 in this case). In the 2^nd^ and 3^rd^ tests (the middle and right panel), the SVR were trained by all glycan occupancy profiles, or PGT121-specific glycan site determinants profiles, respectively. The dash line indicated the expected error based on experimental noises.
