# Supplementary material for: Exploiting glycan topography for computational design of Env glycoprotein antigenicity
Source: PLoS Comput Biol. 2018 Apr 20;14(4):e1006093. doi: 10.1371/journal.pcbi.1006093 (PMC5931682; doi:10.1371/journal.pcbi.1006093)
Supplement: S2 File — (PDF) [file pcbi.1006093.s002.pdf]

>MF535.W0M.ENV.D11

MPMGSLQPLATLYLLGMLVASVLALWVTVYYGVPVWKEATTTLFCASDAKSYKAEAHNIWATHACVPTDPNPREIILE
NVTENFNMWKNNMVEQMHEDIISLWDQSLKPCVKLTPLCVTLNCIEVTVGNTTGTNTTDVGMKNCSFNITTEVRDK
KKQVHALFYKLDVVQIDDNNSTNTSYRLINCNTSAITQACPKVTFEPIPIHYCAPAGFAILKCNDKKFNGTGPCKNVSTVQ
CTHGIKPVVSTQLLLNGSLAEEEIIIRSENLTNNAKIIIVQLNESVTITCTRPYNNTRQSTHIGPGRAYYTTDIIGDIRQAYCTV
NRAEWNRTLQKVSRQLEKYFRNKTIIFANPSGGDLEITTHSFNCGGEFFYCNTSGLFNSTWNHTASTQGSNGNITLNDN
ITLPCRIKQIINMWQGVGKAMYAPPIEGLINCSSNITGLLLTRDGGGNNNTNETFRPGGGDMRDNWRSELYKYKVVRI
EPLGLAPTRAKRRVVEREKRAIGLGAVGGGGSGGGGSGGGGSGLNDIFEAQKIEWHEHHHHHH

>MF535.W0M.ENV.D11|+PGT121

MPMGSLQPLATLYLLGMLVASVLALWVTVYYGVPVWKEANTTLFCASDAKSYKAEAHNIWATHACVPTDPNPREIILE
NVTENFNMWKNNMVEQMHEDIISLWDQSLKPCVKLTPLCVTLNCTEVTVGNTTGTNNLTTTDVGMKNCSFQITTEV
RDKKKQVHALFYKLDVVQIDDNNSTNNTSYRLINCNTSAITQACPKVTFEPIPIHYCAPAGFAILKCNDKKFNGTGPCKN
VSTVQCTHGIKPVVSTQLLLNGSLAEEEIIIRSENLTNNAKIIIVQLNESVTINCTRPYNNTRQSTHIGPGRAYYTTDIIGDIR
QAYCNVTRAEWNRTLQKVSRQLEKYFRNKTIIFNNTSGGDLEITTHSFNCGGEFFYCQTSGLFNSTWNHTASNQTSNN
LTGQITLNDNITLPCRIKQIINMWQGVGKAMYAPPIEGLINCSSNITGLLLTRDGGGNNNTTETFRPGGGDMRDNWRS
ELYKYKVVRIEPLGLAPTRAKRRVVEREKRAIGLGAVGGGGSGGGGSGGGGSGLNDIFEAQKIEWHEHHHHHH

>MF535.W0M.ENV.D11|+PGT128

MPMGSLQPLATLYLLGMLVASVLALWVTVYYGVPVWKEATTTLFCASDAKSYKAEAHNIWATHACVPTDPNPREIILE
NVTENFNMWKNNMVEQMHEDIISLWDQSLKPCVKLTPLCVTLNCIEVTVGNTTGTNNLTNLTTTDVGMKNCSFNITT
EVRDKKKQVHALFYKLDVVQIDDNNSNLTTNTSYRLINCNTSAITQACPKVTFEPIPIHYCAPAGFAILKCNDKKFNGTGP
CKQVSTVQCTHGIKPVVSTQLLLNGSLAEEEIIIRSENLTNNAKIIIVQLNESVTITCTRPYNNTRQSTHIGPGRAYYTTDIIG
DIRQAYCNVTRAEWNRTLQKVSRQLEKYFRNKTIIFAQPSGGDLEITTHSFNCGGEFFYCNTSGLFNSTWNHTASNQTS
NGQITLNDNITLPCRIKQIINMWQGVGKAMYAPPIEGLINCSSNITGLLLTRDGGGNNTNTTETFRPGGGDMRDNWR
SELYKYKVVRIEPLGLAPTRAKRRVVEREKRAIGLGAVGGGGSGGGGSGGGGSGLNDIFEAQKIEWHEHHHHHH

>MF535.W0M.ENV.D11|+PGT121+PGT128

MPMGSLQPLATLYLLGMLVASVLALWVTVYYGVPVWKEANTTLFCASDAKSYKAEAHNIWATHACVPTDPNPREIILE
NVTENFNMWKNNMVEQMHEDIISLWDQSLKPCVKLTPLCVTLNCTEVTVGNTTGTNNLTNNTTTTDVGMKNCSFQI
TTEVRDKKKQVHALFYKLDVVQIDDNNSNLTTNNTSYRLINCNTSAITQACPKVTFEPIPIHYCAPAGFAILKCNDKKFNG
TGPCKQVSTVQCTHGIKPVVSTQLLLNGSLAEEEIIIRSENLTNNAKIIIVQLNESVTINCTRPYNNTRQSTHIGPGRAYYTT
DIIGDIRQAYCNVTRAEWNRTLQKVSRQLEKYFRNKTIIFNQTSGGDLEITTHSFNCGGEFFYCQTSGLFNSTWNHTASN
QTSNNLTGQITNNTNITLPCRIKQIINMWQGVGKAMYAPPIEGLINCSSNITGLLLTRDGGGNNNTTETFRPGGGDMR
DNWRSELYKYKVVRIEPLGLAPTRAKRRVVEREKRAIGLGAVGGGGSGGGGSGGGGSGLNDIFEAQKIEWHEHHHHH
H

>MF535.W0M.ENV.D11|+PGT121-PGT128_1st

MPMGSLQPLATLYLLGMLVASVLALWVTVYYGVPVWKEANTTLFCASDAKSYKAEAHNIWATHACVPTDPNPREIILE
NVTENFNMWKNNMVEQMHEDIISLWDQSLKPCVKLTPLCVTLNCIEVTVGNTTGTNNLTTTDVGMKNCSFQITTEVR
DKKKQVHALFYKLDVVQIDDNNSTNTSYRLINCNTSAITQACPKVTFEPIPIHYCAPAGFAILKCNDKKFNGTGPCKNVST
VQCTHGIKPVVSTQLLLNGSLAEEEIIIRSENLTNNAKIIIVQLNESVTITCTRPYNNTRQSTHIGPGRAYYTTDIIGDIRQAY
CNVNRTEWNRTLQKVSRQLEKYFRNKTIIFNNTSGGDLEITTHSFNCGGEFFYCQTSGLFNSTWNHTASTNGSNNLTG

QITLNDNITLPCRIKQIINMWQGVGKAMYAPPIEGNITCSSQITGLLLTRDGGGNNTNTTETFRPGGGDMRDNWRSEL
YKYKVVRIEPLGLAPTRAKRRVVEREKRAIGLGAVGGGGSGGGGSGGGGSGLNDIFEAQKIEWHEHHHHHH

>MF535.W0M.ENV.D11|-PGT121+PGT128

MPMGSLQPLATLYLLGMLVASVLALWVTVYYGVPVWKEATTTLFCASDAKSYKAEAHNIWATHACVPTDPNPREIILE
NVTENFNMWKNNMVEQMHEDIISLWDQSLKPCVKLTPLCVTLNCIEVTVGNNTTTNNLTNLTTTDVGMKNCSFNITT
EVRDKKKQVHALFYKLDVVQIDNNTSNLTTNNTSYRLINCQTSAITQACPKVTFEPIPIHYCAPAGFAILKCNDKKFNGTG
PCKQVSTVQCTHGIKPVVSTQLLLNGSLAEEEIIIRSENLTNNAKIIIVQLQESVTINCTRPYNNTRQSTHIGPGRAYYTTDII
GDIRQAYCTVNRAEWNRTLQKVSRQLEKYFRNKTIIFAQPSGGDLEITTHSFNCGGEFFYCNTSGLFNSTWNHTASNQT
SNGNITNNTTITLPCRIKQIINMWQGVGKAMYAPPIEGLINCSSNITGLLLTRDGGGNQNTNETFRPGGGDMRDNWR
SELYKYKVVRIEPLGLAPTRAKRRVVEREKRAIGLGAVGGGGSGGGGSGGGGSGLNDIFEAQKIEWHEHHHHHH

>MF535.W0M.ENV.D11|+PGT121-PGT128_2nd

MPMGSLQPLATLYLLGMLVASVLALWVTVYYGVPVWKEANTTLFCASDAKSYKAEAHNIWATHACVPTDPNPREIILE
NVTENFNMWKNNMVEQMHEDIISLWDQSLKPCVKLTPLCVTLNCIEVTVGNTTGTNNLTTTDVGMKNCSFQITTEVR
DKKKQVHALFYKLDVVQIDDNNSTNNTSYRLINCNTSAITQACPKVTFEPIPIHYCAPAGFAILKCNDKKFNGTGPCKNV
STVQCTHGIKPVVSTQLLLNGSLAEEEIIIRSENLTNNAKIIIVQLNESVTINCTRPYNNTRQSTHIGPGRAYYTTDIIGDIRQ
AYCTVNRAEWNRTLQKVSRQLEKYFRNKTIIFNQTSGGDLEITTHSFNCGGEFFYCQTSGLFQSTWNHTASTNGSNNLT
GQITLNDNITLPCRIKQIINMWQGVGKAMYAPPIEGNITCSSQITGLLLTRDGGGNNNTQETFRPGGGDMRDNWRSE
LYKYKVVRIEPLGLAPTRAKRRVVEREKRAIGLGAVGGGGSGGGGSGGGGSGLNDIFEAQKIEWHEHHHHHH
