# Supplementary material for: Exploiting glycan topography for computational design of Env glycoprotein antigenicity
Source: PLoS Comput Biol. 2018 Apr 20;14(4):e1006093. doi: 10.1371/journal.pcbi.1006093 (PMC5931682; doi:10.1371/journal.pcbi.1006093)
Supplement: S1 Table — (PDF) [file pcbi.1006093.s004.pdf]

gp120 ID	clade	name	Tier	Disease status	T/F	cat #	Accession Number
1	A	MK184.W0M.ENV.E4	-	chronic		IT-001-111p	DQ208485
2	A	MK184.W0M.ENV.G3	-	chronic		IT-001-112p	DQ208487
3	A	BL274.W6M.ENV.A3	-	infant infected between birth and 6 weeks post-delivery		IT-001-118p	DQ208499
4	A	ML274.W0M.ENV.F1	-	chronic		IT-001-117p	DQ208497
5	A	92RW020	2_3	seropositive asymptomatic		IT-001-001p	AY669706
6	A	MJ613.W0M.ENV.B1	-	chronic		IT-001-110p	DQ208445
7	A	ML035.W0M.ENV.I2	-	chronic		IT-001-114p	DQ208475
8	A	MI206.W0M.ENV.D1	-	chronic		IT-001-106p	DQ208462
9	A	MG505.W0M.ENV.H3	-	chronic		IT-001-102p	DQ208455
10	A	MF535.W0M.ENV.D11	-	chronic		IT-001-103p	DQ208427
11	B	HcBc2_HBX2	1B	acute	TF	IT-001-0022p	K03455
12	B	SF162	1A	Transmission/Founder		IT-001-0028p	AY669736
13	B	LAI	-			IT-001-138p	A04321
14	B	p1056.TA11.1826	1B	acute	TF	IT-001-122p	EU289186
15	B	AC10.0	2	acute subtype B		IT-001-RB5p	AY835446
16	B	pTHRO4156	2	acute	TF	IT-001-RB10p	AY835448
17	B	pPRB926_04.A9.4239	2	acute	TF	IT-001-133p	EU289197
18	B	YU2	2			IT-001-0027p	M93258
19	B	p1058_11.B11.1550	2	acute	TF	IT-001-123p	EU289187
20	B	p63358.p3.4013	2	acute		IT-001-128p	EU289192
21	C	ZM214M.PL15	2	early stages of seroconvertion		IT-001-RC5p	DQ388516
22	C	TV-1				IT-001-170p	EU855132
23	C	DU172.17	2	early stages of seroconvertion		IT-001-RC2p	DQ411853
24	C	Du156.12	2	early stages of seroconvertion		IT-001-RC1p	DQ411852
25	C	CAP210.2.00	2	early stages of seroconvertion		IT-001-RC12p	DQ435683
26	C	ZM233M.PB6	2	early stages of seroconvertion		IT-001-RC6p	DQ388517
27	C	Du151	2			IT-001-139p	AY043173
28	C	ZM109F.PB4	1B	collected within 3 months of the subject's last seronegative test		IT-001-RC9p	AY424138
29	C	ZM197M.PB7	1B	early stages of seroconversion		IT-001-RC4p	DQ388515
30	C	C.Za.1197MB	-			IT-001-003p	AY463234
31	A	Q769.h5	2			IT-001-0012p	AF407159
32	B	ADA	2			IT-001-0023p	M60472
33	B	JRFL	2	patient with AIDS		IT-001-0024p	U63632
34	B	JRCSF	2	patient with AIDS		IT-001-0025p	M38429
35	B	89.6	2			IT-001-0026p	ABY60458
36	B	R2	-			IT-001-0029p	AF128126
37	B	MN	1A			IT-001-002MNp	AF075720
38	B	Bal	1B			IT-001-002p	M68893
39	CRF07_BC	CN54				IT-001-003CNp	AX149771
40	C	C.ZA.1197MB	-			IT-001-003p	AY463234
41	A	MG505.W0M.ENV.A2	-	chronic		IT-001-101p	dq208449
42	A	MI206.W0M.Env.B1	-	chronic		IT-001-105p	DQ208460
43	A	MJ412.W0M.ENV.B1	-	chronic		IT-001-107p	DQ208435
44	A	MJ412.W0M.ENV.C1	-	chronic		IT-001-108p	DQ208436
45	A	MJ613.W0M.ENV.A2	-	chronic		IT-001-109p	DQ208444
46	A	ML035.W0M.ENV.G2	-	chronic		IT-001-113p	DQ208474
47	A	BL035.W6M.ENV.C1	-	infant infected between birth and 6 weeks post-delivery		IT-001-115p	DQ208480
48	A	ML274.W0M.ENV.B1	-	chronic		IT-001-116p	DQ208493
49	B	p1012.TC21.3257	1B	acute	TF	IT-001-119p	EU289184
50	B	p1006_11.C3.1601	2	acute	TF	IT-001-120p	EU289183
51	B	p1054.TC4.1499	2	acute	TF	IT-001-121p	EU289185
52	B	p1059_09.A4.1460	2	acute	TF	IT-001-124p	EU289188
53	B	p62357_14.D3.4589	2	acute	TF	IT-001-125p	EU289189
54	B	p6240_08.TA5.4622	2	acute	TF	IT-001-126p	EU289190
55	B	p700010040.C9.4520	2	acute	TF	IT-001-129p	EU289193
56	B	p700010058.A4.4357	-		TF	IT-001-130p	EU289194
57	B	p9014_01.TB1.4769	-	acute	TF	IT-001-131p	EU289195
58	B	p9021_14.B2.4571	2	acute	TF	IT-001-132p	EU289196
59	B	pPRB931_06.TC3.4930	2	acute	TF	IT-001-134p	EU289198
60	B	pSC05.8C11.2344	2	acute	TF	IT-001-135p	EU289200
61	B	pSC45.4B5.2631	2	acute		IT-001-136p	EU289201
62	B	pWEAUd15.410.5017	2	acute	TF	IT-001-137p	EU289202
63	A	00KE_KER2018	-			IT-001-149p	AY736810
64	CRF01_AE	CM235	-			IT-001-150p	AF259955
65	CRF01_AE	CM240	-			IT-001-151p	U54771
66	CRF01_AE	90TH_CM244	-			IT-001-152p	AY713425
67	CRF02_AG	98US_MSC5007	-			IT-001-153p	AY736840
68	CRF02_AG	01CM_0002BBY	-			IT-001-154p	AY736843
69	CRF02_AG	01CM_1475MV	-			IT-001-155p	AY736842
70	CRF07_BC	CN54	-			IT-001-160p	AX149771
71	CRF07_BC	Consensus	-			IT-001-163p	AY008716
72	CRF01_AE	A244	-	chronic		IT-001-164p	AAW57760
73	CRF07_BC	CH181	2			IT-001-166p	EF117259
74	CRF01_AE	Consensus	-			IT-001-168p	AF259955
75	B	BL10	-			IT-001-172p	
76	C	001428-2.4	2			IT-001-174p	EF117266
77	A	Q461.d1				IT-001-175p	AF407155
78	A	Con_A1	-			IT-001-CONA1p	
79	A	Con_A2	-			IT-001-CONA2p	
80	B	Con_B	-			IT-001-CONBp	
81	C	Con_C	-			IT-001-CONCp	
82	C	Con_C	-			IT-001-CONCp	
83		Con_of_Cons	-			IT-001-CONp	
84	B	pCAAN5342	2			IT-001-RB11p	AY835452
85	B	SC422661.8	2	acute		IT-001-RB12p	AY835441
86	B	6535	1B	acute		IT-001-RB1p	AY835438
87	B	QH0692	2	acute		IT-001-RB2p	AY835439
88	B	PVO	3	acute		IT-001-RB3p	AY835444
89	B	TRO	2			IT-001-RB4p	AY835445
90	B	pWITO4160	2	acute	TF	IT-001-RB6p	AY835451
91	B	pTRJO4551	3	acute	TF	IT-001-RB7p	AY835450
92	B	pREJO4541	2	acute	TF	IT-001-RB8p	AY835449
93	B	pRHPA4259	2	early subtype B	TF	IT-001-RB9p	AY835447
94	C	ZM135M.PL10a	2	collected within 3 months of last seronegative test.		IT-001-RC10p	AY424079
95	C	CAP45.2.00	2	acute		IT-001-RC11p	DQ435682
96	C	Du422.1	2	early stages of seroconvertion		IT-001-RC3p	DQ411854
97	C	ZM214M.PL15	2	early stages of seroconvertion		IT-001-RC5p	DQ388516
98	C	ZM249M.PL1	2	acute	TF	IT-001-RC7p	DQ388514
99	C	ZM53M.PB12	2	within 3 months of the subject's last seronegative test		IT-001-RC8p	AY423984
